# Eye care interventions that reduce access inequities for women, rural residents and older people in low-middle-income countries: a scoping review

**DOI:** 10.3389/fpubh.2025.1578848

**Published:** 2025-06-23

**Authors:** Magnolia Cardona, Kennedy Alwenya, Atiq ur Rehman, Sarah Olalo, An Thai, Mediya Rangi, Yadira Perez, Ling Lee

**Affiliations:** ^1^The Fred Hollows Foundation, Melbourne, VIC, Australia; ^2^School of Population Health, The University of New South Wales, Sydney, NSW, Australia; ^3^Institute for Evidence-Based Healthcare, Bond University, Gold Coast, QLD, Australia; ^4^The Fred Hollows Foundation, Nairobi, Kenya; ^5^The Fred Hollows Foundation, Islamabad, Pakistan; ^6^The Fred Hollows Foundation, Da Nang, Vietnam; ^7^School of Optometry and Vision Science, The University of New South Wales, Sydney, NSW, Australia; ^8^National Vision Research Institute, Australian College of Optometry, Melbourne, VIC, Australia

**Keywords:** inequities, eye care barrier, blindness, low-middle-income, screening, cataract, diabetic retinopathy, surgery

## Abstract

**Introduction:**

Women, older people and rural residents in low-middle-income settings are mainly impacted by the economic and psychosocial consequences of preventable blindness from undiagnosed and untreated cataracts diabetic retinopathy.

**Methods:**

This review of PubMed 2002-2023 and the grey literature aimed to identify strategies effective in reducing access inequities to eye health screening and treatment for the above vulnerable groups.

**Results:**

Thirty-nine publications from 16 countries were included. Fifteen focused on cataract, 17 on diabetic retinopathy, and seven on general ophthalmology. This article focuses on the twenty-four studies of moderate or high quality. Rural residents were more likely to benefit (16 studies) while direct effectiveness among women were reported in seven studies. Only three studies reported actual benefits for older people. Outreach services and teleophthalmology were effective interventions increasing screening attendance and referral rates for women and rural residents. Health financing to enhance cataract surgery acceptance and actual surgical rates reported effectiveness for rural residents but showed only modest improvements. Digital technology improved overall appointment uptake and referral adherence for rural residents but not significantly for women. Teleophthalmology was successful in building local capacity for accurate diagnosis but its impact on referral compliance was not demonstrated. Limited evidence was found for the effectiveness of health education alone to boost screening attendance for either subgroup.

**Discussion:**

The evidence for effectiveness in reducing inequities is not always direct, uses mixed outcomes, and had heterogenous designs. Yet, the results of the higher quality publications in this review indicate modest improvements worth pursuing further.

**Systematic Review Registration:**

https://osf.io/yr7tg/files/osfstorage?view_only=968ba9e8c910470ca227dcdb0da3cda8.

## 1 Introduction

Vision impairment and blindless have profound and widespread implications for many aspects of life, general health, and a sustainable economy (1). In 2020, an estimated 1.1 billion people lived with either distance vision impairment or had uncorrected near vision impairment worldwide and 43 million were blind. More than 90% of vision loss is preventable and/or treatable with existing cost-effective interventions (1). As the global population grows and gets older, age-related cataract, diabetic retinopathy, macular degeneration, and glaucoma put more people at risk of blindness (2). Moreover, vision loss is not evenly distributed across all countries. Of those impacted, 90% live in low-income and middle-income countries (2) where health inequities result in a greater disease burden on young children, women, older people, rural populations, and ethnic minorities due to limited access to essential healthcare (1).

The World Health Organization recognizes that eye care must be an integral part of universal health coverage with effective integration to contribute to achieving the UN Sustainable Development Goals (SDG) (3). Poor eye health has long-term negative impact on quality of life, including achievements in education, employment and economic participation. Reduced mobility; diminished mental and emotional wellbeing; increased risk of dementia, falls, and road accidents, all lead to increase in demand on family members to fulfill the role of carers (1). This is a role that is disproportionately and predominantly expected of women and girls which in turn further perpetuates inequities for women (4–6). Indeed, the financial implications of vision loss extend beyond the individual to the families and communities. To achieve the SDGs, a coordinated effort with fresh and innovative approaches is required to prevent avoidable blindness, particularly cataract and diabetic eye diseases which are among the leading causes of vision loss (1, 7).

The prevalence of vision impairment is higher in girls and women than in boys and men, especially in low- and middle-income countries. Of the 43 million people who are blind, 24 million (55%) are women, and of those with moderate to severe, mild, and near vision impairment, 585 million (55%) are women (2). This disparity is due to biological factors (the average life expectancy of women is longer than for men and are at more risk of developing certain eye conditions including cataract) and social influences as gender-based discrimination leads to gender-based disparities in access to education, healthcare, and resources (2, 3).

A recent study of cataract surgery contends that the gender disparity in eye disease/service intertwined with social, economic, and cultural differences between men and women, is still prevalent in South Asian society (8). The interconnectedness between the enforcement of gender norms in society and the external dimensions that reduce women’s ability to seek healthcare in general and eye treatment have been reported in various countries (9). Despite this, policy change to reduce inequities of access has been slow (10) and a solution to address this human right gap is overdue (1, 11).

Additionally, rural populations experience greater barriers to accessing eye care due to long travelling distances and inadequate transport infrastructure including accessible roads, as well as family obligations, lack of knowledge of asymptomatic eye illnesses and economic reasons (12, 13). Limited human resources and equipment in rural areas also lead to unmet eye care needs in these settings with very limited number of health workers with adequate training in eye care (14). As a result of these factors, there is a lower cataract surgical coverage and higher prevalence of cataract are commonly reported in rural areas.

### 1.1 Objectives

This review aimed to answer the following research questions:

1. What are the effective interventions that enhance access to eye health screening and eye disease treatment completion for diabetic retinopathy and cataract among women and rural residents in low-resource settings?
2. What is the extent and sustainability of that effectiveness?
3. What are the most effective components that can inform future sustainable interventions to enhance access to eye health care?
4. What are the success factors for scalability and/or sustainability in low- and middle-income countries (LMICs) as mapped to the RE-AIM knowledge translation framework? (15).

## 2 Methods

We conducted a scoping review of publications indexed in the PubMed database between 2002 and 2023 without language restrictions and from the gray literature of targeted sites (International Agency for the Prevention of Blindness, World Health Organization, United Nations General Assembly, Fred Hollows Foundation, NHS reports on development initiatives) as the most relevant sources to identify eye interventions implemented in low-mid resource settings (according to World Bank knowledgebase) (16). The target population was therefore adult residents in those countries who were either screened or treated for the target conditions of interest to our organization: cataract and diabetic retinopathy. For the purpose of this review, eligible interventions were new strategies, practices, technological advances, policies or incentives tested in real life conditions. We excluded study designs that did not report the evaluation of an intervention. Effectiveness was defined as any measure of impact that actually or potentially enhanced access to eye care for our sub-populations of interest, even if these were not the main aim of the eligible publications. Qualitative and mixed methods studies of perceived effectiveness without estimates were excluded from this manuscript and will be reported in a separate manuscript.

Eligible publication types were randomized or non-randomized controlled trials, cohort studies, before-after studies testing the introduction of an intervention, retrospective analysis of a service database with analysable post-intervention outcomes, comparative accuracy studies of technology with potential to reach rural residents (e.g., telehealth, mobile health), and descriptive analyses of health financing modalities or partial subsidy policies to reduce inequities of access.

Outcomes of interest included but were not limited to quantitative estimates of: change in screening attendance; increased referral and follow-up; treatment completion rates; changes in eye care service use post-intervention; acceptance of surgical procedures; response to health financing changes such as surgical acceptance rates, and accuracy of health worker detection of anomalies versus specialist diagnosis. We embraced heterogeneity of outcomes due to the anticipated variety of strategies and study designs as long as they presented measurements, and chose the REAIM (Reach, Adoption, Implementation and Maintenance) framework domains to ascertain effectiveness given its widespread use, accessibility and relevance (15). We did not plan or attempt to contact authors for clarification or completion of data items.

The search strategy included combining four concepts: eye care, inequality, LMIC and effectiveness terms. The details are presented in Supplementary Table S1.

The study protocol was registered on OSF.^1^

### 2.1 Data extraction and synthesis

Reviewers (KA, SO, AR, AT, LL) individually screened titles and abstracts with a senior reviewer (MC) checking all potentially includable and 10% random samples of excluded from each screener based on title and abstract. Once agreed, paired reviewers screened full texts of all eligible and resolved discrepancies by discussion. One screener (LL, KA, AR, AT, SO, MR) individually undertook data extraction and the lead author (MC) checked accuracy using pre-defined structured tables for study characteristics, intervention description and results. Screeners manually searched reference lists of reviews for relevant primary studies potentially amenable to full text screening. The Template for Intervention Description and Replication (TIDIeR) (17) guided intervention descriptions. Paired independent reviewers (LL, MR) assessed risk of bias following a pre-defined assessment tool (quality score calculated by adding 1 point per assessment criteria, with 1 being the lowest quality and 12 the highest). The criteria catered for different study types, based on modification of existing tools (18) (Supplementary Tables S2.1, S2.2) and discrepancies were resolved by discussions with a third reviewer (MC). If risk of bias was not fully assessable due to lack of information (6 studies out of the 39 included), the results of those publications were presented in appendices.

Driven by our duty of care in preventing misleading designs or doubtful quality reports or unsupported conclusions from influencing public health practice, investigating effectiveness, sustainability and scalability were synthesized and analysed only from the publications with moderate high quality. To reduce the risk of erroneous recommendations for research translation, only outcomes from the high and moderate quality studies (i.e., scores of 9–12, and 7–8 respectively) were mapped to the REAIM (Reach, Efficacy, Adoption, Implementation, and Maintenance) framework (19) when feasible (i.e., if domains reported).

Analysis is purely descriptive with summary tables of relevant outcomes and an assessment (effectiveness Yes/No/not reported) and description of intervention characteristics presented for publications in descending order of quality score. When studies were conducted solely in rural areas, access to eye services was assumed for this subpopulation regardless of whether there was a comparison group. In addition to reporting our target subpopulation outcomes, we found some details of access for older people and added this aspect to all our results tables. In cases of overall intervention effectiveness without reporting of gender or age differences our tables state potential effectiveness (NR/P) if the assumption is not contentious. For instance, when diagnostic accuracy by trained rural health workers was overall comparable to that of specialists, but there were no results for women or older people, then we assume potential effectiveness for these groups. However, if portable technology like smartphones is compared to usual assessment with specialist equipment but the people testing accuracy are not community health workers but city officers such as ophthalmologists or trained vision technicians, then the assumption of accuracy/effectiveness for rural areas does not hold. This is because the health workers receive comparatively minimal training, have competing health tasks to undertake, and lower diagnostic sensitivity for referral decisions than urban trained officers (20).

## 3 Results

**Figure 1:**
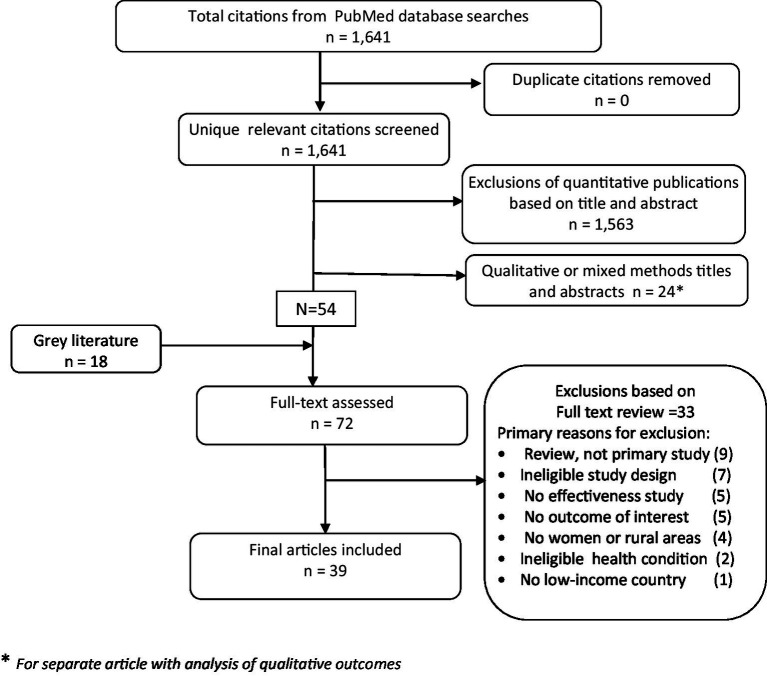
PRISMA diagram of screening and extraction process.

Of 1,641 titles in PubMed, and 18 in the gray literature, 72 full texts were screened and 39 publications from 16 countries met the eligibility criteria, mostly (77%) released in the past decade. The main reasons for exclusion were review papers -which we further examined to select relevant primary studies from- and ineligible study designs such as prevalence or cost-utility analyses (Figure 1 and Supplementary Table S3).

### 3.1 Study characteristics

Of the included publications (i.e., peer-reviewed or reports), 15 focused on cataract, 17 on diabetic retinopathy, and seven on general ophthalmology, with a clear predominance of studies from India and China but with representation from four continents (Table 1). Of the 39 included studies some targeted overall population rather than specific subgroups and some targeted more than one subgroup. Target groups most frequently captured by the initiatives were women and residents of rural areas, whereas only 6 studies directly reported results for older patients or having older populations as their target (note that these numbers of studies regardless of interventions effectiveness). The most common study types for effectiveness investigations were prospective cohorts, and a variety of non-randomized designs, whereas only a handful being randomized controlled trials contributed to this evidence gathering. Thirteen cross-sectional studies of either comparative accuracy of assessment between remote health workers and urban specialists (teleophthalmology) or description of outreach interventions were also included due to their potential for reducing access inequities.

**Table 1:** Eligible study characteristics (*n* = 39).

Feature	Description	No. of studies*
Year of publication	2005–2014	9
	2015–2023	30
Condition addressed	Cataract	15
	Diabetic retinopathy	17
	General ophthalmology	7
Countries*	India	10
	China	8
	Other Asia: Vietnam, Myanmar, Nepal, Bangladesh, Malaysia, Pakistan, Iran	11
	Africa: Uganda, Rwanda, Nigeria, Tanzania	6
	South America: Brazil, Argentina	3
	Timor Leste	1
Beneficiary population captured	Overall population (no specific subgroup reported)	6
	Women^§^	32
	Rural residents^§^	22
	Older people^§^	6
Study methods	Cross-sectional	13
	Prospective cohort	9
	Randomized controlled trial	6
	Retrospective analysis	6
	Before-after design	4
	Modeling	1

*Number of studies can add to more than 39 as some studies captured more than one population subgroup and some countries had more than one eligible study. ^§^Based on reported distribution of participants even if not the primary target group.

For details of study characteristics at the individual level see Supplementary Table S4 and for components of each intervention see Supplementary Tables S5.1–S5.5.

### 3.2 Risk of bias assessment

The quality score of the 33 studies assessed, revealed that only 14 studies (42.4%) were classified as high quality (21–33) that is, scores of 9+; 10 as moderate quality defined as score of 7–8 (30.3%) (34, 35) and 9 as low quality (27.3%) or scores of 6 and below. Six non-peer reviewed reports were not assessed for bias due to lack of information on several quality criteria (15.4% of all eligible studies). Studies generally specified inclusion criteria, had acceptable case definitions, used some form of validated diagnostic criteria, followed a pre-specified analysis plan and yielded conclusions supported by findings. However, in addition to most studies not being effectiveness trials, among the 33 assessed peer-reviewed publications, the most common reasons for risk of bias were likelihood of attrition after recruitment, uncertainty about standard data extraction, and lack of adjustment for potential confounders. These flaws may skew results as those lost to follow-up may have different risk factors or other socio-economic characteristics impacting responses to treatment offers, and lack of adjustment could lead to misinterpretation. The next common risk of bias was potential lack of representativeness due to absence of random selection or sampling frame flaws (Supplementary Tables S2.2, S2.3). This is common in real-world studies due to convenience but needs to be considered as potentially impacting on generalizability.

### 3.3 Intervention effectiveness

**Figure 2:**
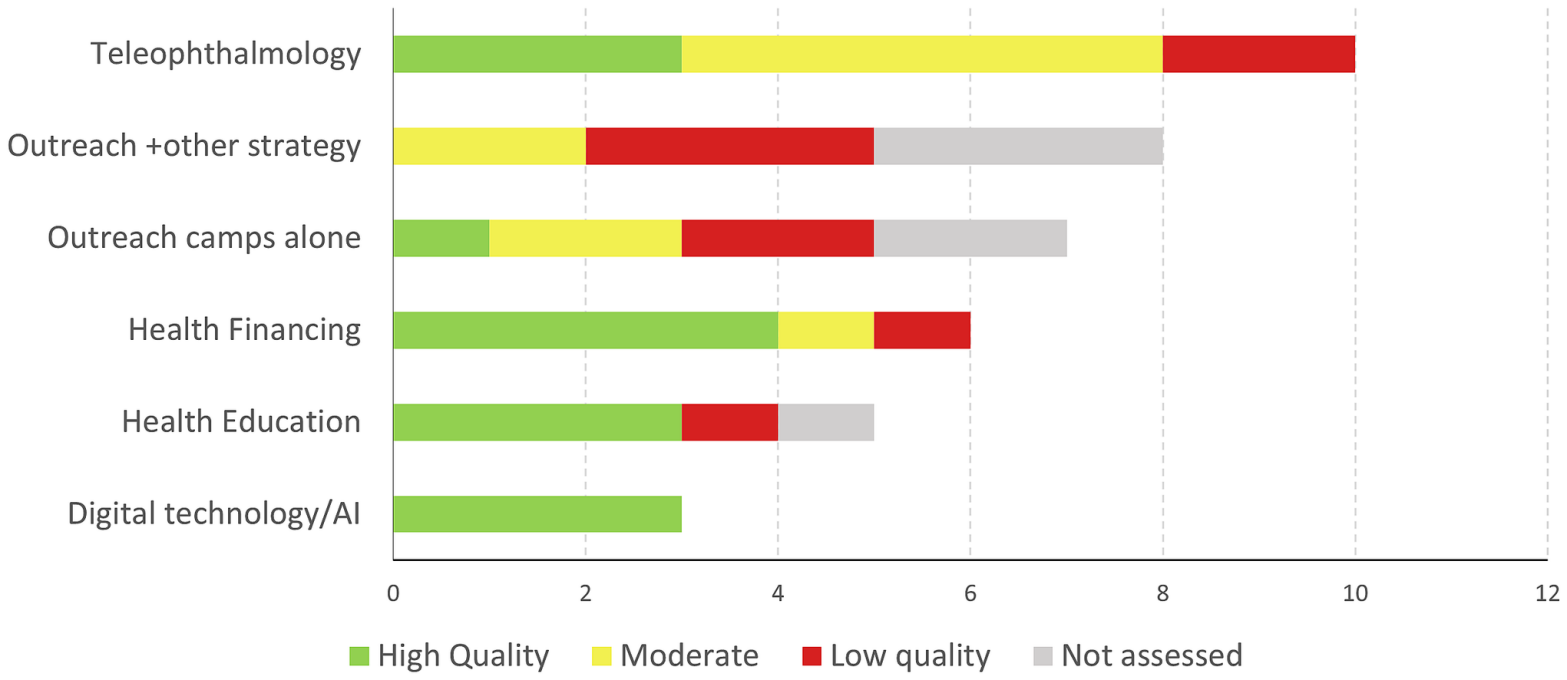
Intervention types of included studies by quality.

Five main strategies dominated the attempts to reduce access inequities: teleophthalmology accuracy trials, outreach initiatives, health financing modalities, education campaigns, and digital technology/artificial intelligence testing (Figure 2).

To inform evidence-based potential for applicability or replication by others in similar settings, we present effectiveness results by type of intervention in descending order of quality score out of a maximum of 12. Tables present the three RE-AIM components available to assess: Reach, Effectiveness and Maintenance.

#### 3.3.1 Accuracy studies of teleophthalmology

Ten studies of teleophthalmology interventions were conducted via non-medical technicians and image graders with real-time specialist advice in an urban location (20, 23, 30, 34, 36–41). Our focus will be on only eight: three studies of high (23, 30, 41) and five of moderate quality (20, 34, 36, 39, 40). Generally, the teleophthalmology studies were proof-of-concept studies that compared the accuracy of assessment in terms of percentage agreement of technician’s grading on the basic presence or absence of condition and need for further referral, or sensitivity/specificity of technician’s classification with the specialist’s opinion (Table 2). The capability of teleophthalmology to potentially reduce inequities varied from highly promising to modest performance.

**Table 2:** Teleophthalmology accuracy and potential effectiveness (*n* = 8).

Author/year country	Target condition	Quality score/12	Reach: did it include intended group or enhance access for…? (Yes/No/Potentially/NR)			Effectiveness results – relevant outcomes	Maintenance
			Women	Rural	Older		
Bob-Semple 2017 Uganda (41)	DR	10	NR	N	NR	-For DR diagnosis by ophthalmologist resident: Sensitivity: 70% (95% CI, 47.1–86.8%) Specificity: 94% (95% CI 89.3–96.9) -For grading non-proliferative DR Sensitivity: 60.9%; Specificity: 94.3% -proliferative DR Sensitivity: 100%; Specificity: 99.4%	NR
De Araujo, 2021 Brazil (23)	GO	10	**Y**	**Y**	NR	Remote team reached **64.4%** women. **94.8%** concordant classification between specialist and teleophthalmology team led by nurse technician; No statistical difference in image suitability between remote team **(93.7%)** and ophthalmologist **(94.4%)** ***p*** **= 0.512**	NR
McKenna, 2018 China (30)	DR	9	NR/P	**Y**	NR/P	-Non-medical graders Any DR: Sensitivity 94%; Specificity 91%, **Kappa 0.85,** ***p*** **< 0.001** DR requiring treatment: Sensitivity 88%; Specificity 99%, **Kappa 0.87**, ***p*** **< 0.001** -Trained rural ophthalmologists Any DR: Sensitivity 95%; Specificity 59%, **Kappa 0.52,** ***p*** **< 0.001** DR requiring treatment: Sensitivity 66%; Specificity 91%, **Kappa 0.48,** ***p*** **< 0.001**	NR
Yusuf 2022 Uganda (34)	DR	8	Y	NR/P	NR/P	Sensitivity of PEEK retina for DR diagnosis by was **84%** (95% CI 70.9–83.5), and specificity **79.9%** (95% CI 76–83.5) **74.5%** of participants were female	NR
Queiroz, 2020 Brazil (40)	DR	7	P	N	N	In an urban setting, **70%** of exams were gradable for DR, **81.2%** of exams enabled clinical decisions M/F referable DR (46%/54%) and M/F non-referable DR (23.8/76.2%)	NR
Collon, 2020 Nepal (36)	C, RE, GO	7	N	**Y**	N	Diagnostic assessment worked best for cataract (**kappa = 0.732**, 95% CI 0.65–0.81) and worst for optic nerve pathology (k = 0.057, 95% CI −0.03 to −0.14) Agreement on need for surgery was modest (k = 0.623, 95% CI 0.49–0.75) but agreement on need for referral was too low (k = 0.12, 95% CI 0.0–0.24) Technicians were more likely to diagnose “No Abnormality Detected” than ophthalmologists	NR
Das, 2019 India (20)	GO	7	NR	**Y**	NR	53.7% of cases seen by technician required crosschecking of diagnosis, 20% needed confirmation of medical management, and 16.5% queried surgical referral but only 0.6% needed it. **73%** of the patients were able to receive timely intervention.	NR
Ortiz-Basso, 2019 Argentina (39)	GO	7	**Y**	**Y**	NR	Annual rate of fundoscopy among people with diabetes (70% women) Overall in rural areas: **Before: 39.3% (30.9–48.3)** **After: 78.6% (70.4–85.1)** ***p*** **< 0.001** Urban area fundoscopy: 55.7% (46.3–64.1)	NR

C, Cataract; DR, Diabetic retinopathy; GO, General ophthalmology; NR, Not reported; P, Potentially.

A cross-sectional analysis comparing smartphone photography with the gold standard ophthalmoscopy for DR in Uganda found suboptimal sensitivity for diagnosis, thus it was not recommended for routine use despite high performance on grading (41). Findings in an urban center in Brazil analysing randomly selected fundus images acquired by nurse technicians in remote locations indicated they were as suitable for diagnosis as those taken by ophthalmologists and condition classification by the nurses was over 90% (23). These findings, from mostly female patients attending, provided strong evidence of adequacy of nurse technicians in capturing remote patients with sight-threatening conditions requiring referral and management. In China, fundus photography interpretation of a cohort of rural residents with diabetes found that when compared with a rural senior specialist, trained ophthalmic nurses had higher accuracy in detecting any DR and referable DR than rural ophthalmologists (30). While this is promising to enhance access to diagnosis and referral within existing resources, the authors highlighted the need for enhanced training for rural doctors.

Among the moderate quality studies, by contrast, a Ugandan comparison of portable eye examination kit technology with standard ophthalmic fundus camera reported marginally higher sensitivity and lower specificity for DR diagnosis but recommended it as suitable for low-income settings based on overall performance, low cost and portability (34). An intervention in an urban center in Brazil examined performance of nurses without previous procedural experience using smartphone retinal photography by following a protocol under ongoing remote specialist feedback (40). The results appeared promising in terms of ability to grade and potential for reducing travel requirements for patients but were not statistically analysed for inter-rater reliability (36). Likewise, an Indian teleophthalmology initiative with eyeSmart EMR and real-time access to ophthalmologist revealed that virtual consultations largely assisted in referral or treatment decisions but did not assess technician performance against a gold standard (20). A Nepalese study attempted the mobile device photographic assessment but found only modest performance by ophthalmic technicians diagnosing cataracts and optic nerve conditions and had poor agreement on interpretation of the need for referral (36).

Finally, a before-after telephone study where patients self-reported undergoing fundoscopy compared urban and rural areas after a teleophthalmology program in Argentina. Results suggested a significant increase in screening for later specialist assessment but again, no data were presented on actual diagnosis or management (39).

#### 3.3.2 Effectiveness of outreach strategies

Over 38% (15/39) of the studies included some form of outreach camps either alone (32, 35, 42–46) or in combination with other intervention components (47–53). However, only one was of high quality (32) and four of intermediate quality (35, 43, 49, 50), three reporting outreach alone for either cataract or DR (32, 35, 43) and two implementing outreach with teleophthalmology (49, 50).

As seen in Table 3, an outreach intervention conducted by ophthalmologist/ophthalmic nurse teams in rural China was successful at identifying and reaching older people and more women with cataract from low socio-economic position than a static clinic in urban areas (32). Screening rates and willingness to undergo low-cost surgery were high suggesting potential to overcome access inequities for women and rural residents but they did not match the levels achieved in static urban clinics where willingness to pay was higher. Also in China, a prospective outreach DR screening conducted by non-medical graders in rural primary care centers reached significantly more women with sight-threatening retinopathy previously diagnosed with diabetes than passive case detection at secondary-level hospitals, but did not report on subsequent treatment or completion rates (35).

**Table 3:** High and moderate quality outreach initiatives (*n* = 5 studies).

Author/year, country	Target condition	Quality score/12	Reach: did it enhance access for…? (Yes/No/NR)			Effectiveness results – relevant outcomes	Maintenance
			Women	Rural	Older		
Zhang 2010 China (32)	C	9	**Y**	**Y**	N	Willingness to undergo low-cost cataract surgery outreach screening vs. static clinic: **78.3% vs. 92.5%,** ***p*** **< 0.001** Women participants: outreach **74.2%** vs. static clinic **54.3%**, ***p*** **= 0.002** Mean age: outreach 74.9 years vs. static clinic 73 years, *p* = 0.09	NR
Xiao 2022 China (35)	DR	8	**Y**	**Y**	**Y**	Reached women (**62.3%** outreach screening vs. **50.8%** passive case detection, ***p*** **= 0.006**). Reached older people aged ≥65 years (**49.5%** outreach vs. **37.8%** passive, ***p*** **= 0.03**) years (**49.5%** outreach screening vs. 37% passive case detection, ***p*** **= 0.03**)	NR
Mohan 2012 India (50)	DR	7	**Y**	**Y**	NR	Attendance rate **86.5%** 4.9% had severe DR requiring laser treatment. **55.8%** of screened were women **95%** of the diabetes complications could be attended locally.	Y
Amritanand 2018 India (43)	GO	7	N	**Y**	N	**97.6%** of those presenting to the special clinic were correctly referred by the CHWs. **39.6%** increase in uptake of eye services compared to previous year but low follow-up rate (15% only)	NR
Katibeh 2020 Iran (49)	GO	7	N	**Y**	N	OR (95% CI) Eye care utilization: after home mobile screening trial compared to before: mHealth **1.7 (1.2–2.4)**, ***p*** **= 0.001** after conventional screening at primary care center 1.2 (0.8–1.8) *p* = 0.37 Control/no proactive screening 0.07 (0.05–0.11), ***p*** **< 0.001** No gender or age differences *p* > 0.05	NR

C, Cataract; DR, Diabetic retinopathy; GO, General ophthalmology; NR, Not reported.

In India, multidisciplinary outreach teams including an ophthalmologist, allied health workers, technicians and village workers combined education, eye screening, imaging and laboratory testing and achieved high referral attendance rates at a rural hospital with over half of attendees being women whose complications were managed on-site (50). Another Indian initiative trained health workers to identify general visual problems and encouraged impaired patients to attend a temporary ophthalmic clinic where specialists examined and referred them to the base hospital. While referral appropriateness by health workers was high, patient adherence to follow-up presentation was low despite reminders (43). In Iran a cluster RCT where screening was driven by primary health care workers and ophthalmic technicians, compared screening with smartphone in the patient’s home with conventional screening at primary care centers, both offering image reading by a specialist. Increases in eye care utilization after home mobile screening were significantly higher in the rural districts but women were as likely as men to participate (49).

#### 3.3.3 Health financing

The intervention studies with the highest level of quality were four conducted in Asia and one in Africa to enhance surgical rates (22, 24, 25, 33, 54). A randomized trial in China evaluated four options (Table 4) of varying degrees of cataract surgery subsidy for rural residents and found relatively small increases in surgical uptake mostly by men despite the intervention addressing additional transport barriers (33). A modeling study of hypothetical programs to overcome access barriers combined real patient survey data, reports from the local literature and stakeholder-confirmed assumptions in Vietnam. The authors found that eliminating direct surgical costs and out of pocket expenses for men and women with cataracts would amount to <0.01% of the total national healthcare budget and could avert disability, particularly for women (24). In China, a retrospective case series saw an increase in cataract surgery rates in rural areas higher than in urban areas over a 6-year period after the introduction of an additional health insurance coverage reform for rural residents (22). Also in China, a multicomponent intervention of public campaign, phone reminder and compensated travel expenses aimed to re-capture patients who did not return for surgical follow-up at 3 months. The initiative achieved increased attendance, particularly for women, younger people, those living closer to the hospital and those satisfied with the surgical outcome (25). In Tanzania, a follow-up study of patients who refused cataract surgery were offered counselling and a waiver of the surgical cost if they proved to be poor. Despite these efforts few sought or used the waiver due to fear of surgery or distrust in the health system (54).

**Table 4:** Effectiveness of health financing approaches (*n* = 5).

Author/ year country	Target condition	Quality score/12	Reach: did it include the intended group or enhance access for…? (Yes/No/Potentially)			Effectiveness results – relevant outcomes	Maintenance
			Women	Rural	Older		
Zhang 2013 China (33)	C	11	N	**Y**	N	Cataract surgery acceptance rate: Grp 1 (low-cost surgery): 15.1% Grp 2 (free surgery+ reminder): **29.1%** Grp 3 (free surgery + transport refund):31.1% Group 4 (free surgery + trip arranged): **28.0%** **1 vs. 2, *p* = 0.027**; 2 vs. 3, *p* = 0.7683; 3 vs. 4, *p* = 0.640; 2 vs. 4, *p* = 0.869 Accepting surgery was **not** associated with age, education level or presenting visual acuity (all *p* > 0.05). More men than women accepted, *p* = 0.03	NR
Essue 2020 Vietnam (24)	C	11	P	NR	NR	Modeling Programme eliminating out of pocket expenses in addition to surgical subsidy costs $1,641,835 / year. Both <0.01% total national health care spending. **Women would avert 4 times as many DALYs as men**	NR
Chen 2011 China (22)	C	10	NR/P	**Y**	NR	The total number of cataract surgeries had increased each year from 169–630 in rural, and from 1,095–2,523 in urban areas as had the proportion of patients with health insurance from **7.7–29.7% in rural** and from **36.3–45.3% in urban areas**	NR
Huang 2012 China (25)	C	9	**Y**	**Y**	N	**66.0% of surgical follow-up defaulters in rural area attended** a compensated examination, mostly younger (***p* = 0.002) and women 76.2%** vs. 23.8%, ***p* = 0.017**. Only 39.9% knew they had to return (23% with higher income *p* = 0.037 had previously returned for uncompensated follow-up)	NR
Kessy 2007 Tanzania (54)	C	7	N	NR	NR	79% gave cost as reason for defaulting. No age or sex differentials (*p* > 0.05) 20% returned with money and 2.5% came back with waiver from village leader 17% did not seek waiver due to disbelief in the health system	NR

C, Cataract; NR, Not reported; P, Potentially.

#### 3.3.4 Health education

Five studies investigated the effectiveness of health education approaches in five countries in Asia and Africa but only three were of sufficient quality to inform practice (Table 5). In Bangladesh, a cohort of non-compliant patients with DR received basic eye screening followed by a referral and intensive personalized diabetes/DR health education and telephone reminders to encourage attendance to treatment. This led to an overall significantly greater proportion complying with referrals than the usual care group, but the more educated people and men were more likely than women to attend (26).

**Table 5:** Effectiveness of health education strategies (*n* = 3).

Author/year country	Target condition	Quality score/12	Reach: did it include the intended group or enhance access for…? (Yes/No/Potentially)			Effectiveness results – relevant outcomes	Maintenance
			Women	Rural	Older		
Khair 2020 Bangladesh (26)	DR	12	N	NR	N	Overall referral compliance for personalized health education group vs. standard care group: **64.3% vs. 28.2%, *p* < 0.001 [OR] 4.73 95% CI 2.87–7.79** Gendered referral compliance after intervention Men vs. women (54.4% vs. 45.6% *p* = 0.061) Compliance by age groups did not differ, *p* = 0.476; and compliance among 60 + year-olds was 28.7% vs. non-compliance 33.1%	NR
Ko 2021 Myanmar (27)	C	10	NR/P	NR	**Y**	Older people accessed eye care services after education: Intervention vs. control: **85.7% vs. 39.3% *p* < 0.001** Knowledge scores improved ***p* < 0.001** Intervention group: 10.7 ± 1.2 **Control** group: 7.7 ± 2.3	NR
Liu 2012 China (28)	C	9	N	**Y**	N	An increase in Surgery acceptance in both rural groups: Intervention group (31.1%) vs. Control group (34.2%), *p* > 0.50. Women OR 1.02, 0.76–2.08 Younger age OR 0.99–1.04 Higher income (**OR 1.27, 1.09–1.47**) and anticipated loss of income (**OR 1.36, 1.01–1.83**) were significant predictors	NR

DR, Diabetic retinopathy; C, Cataract; NR, Not reported; P, Potentially.

In Myanmar, a comprehensive door-to-door monthly education of older people with cataracts supplemented by videos, handouts, T-shirts, calendars and posters had a large 6-month post-intervention impact on access to eye care services when compared to a single information session in the control group (27). Importantly, the social determinants of access to healthcare such as transport, distance, convenience, affordability and social support were no different between groups at baseline (*p* > 0.05 for all).

A randomized trial of an education campaign in rural China used a 5-min information video on family impact of cataracts and the process from hospital arrival to post-operative discharge followed by 5-min scripted counselling on surgery and its cost. Unfortunately, it achieved similar increases in surgical acceptance there were no age or sex differences as in the control group who received no education, video or counselling before surgery (28).

#### 3.3.5 Other digital and AI technology

Three good quality studies of digital interventions such as automated SMS reminders or testing of AI supported screening automation conducted in Africa and Asia aimed to test the sensitivity and specificity of devices or the effectiveness in enhancing referral uptake (21, 29, 31). In rural China, an RCT for people with diabetes compared two automated SMS reminders within the week of the appointment to scheduling without reminders. The digital approach significantly improved appointment attendance but over half the patients still failed to comply with their appointment (21) (Table 6).

**Table 6:** Effectiveness of other digital or AI technology (*n* = 3).

Author/year country	Target condition	Quality score/12	Reach: did it include the intended group or enhance access for…? (Yes/No/Potentially)			Effectiveness results – relevant outcomes	Maintenance
			Women	Rural	Older		
Chen 2018 China (21)	DR	11	N	**Y**	N	Appointment attendance: **42.9%** intervention vs. **14.0%** in control group, ***p* < 0.001**. Likelihood for **rural** in intervention: (**RR 3.04, 95% CI, 1.73–5.33**) Male/female attendance (RR 0.68 95% CI, 0.41–1.11, *p* = 0.124) Age, and baseline patient satisfaction score were not associated with appointment attendance (*p* > 0.05).	NR
Mathenge 2022 Rwanda (29)	DR	10	N	**Y**	Y	Referral adherence: **51.5%** intervention vs. **39.6%** control group. Intervention (**OR 1.73 95% CI, 1.04–2.87**) Males (OR 2.08 95% CI, 1.22–3.54, *p* = 0.007) Rural residents’ adherence (**OR 1.77 95% CI, 1.05–2.99, *p* = 0.033**) Older participants (**OR 1.04 95% CI, 1.02–1.05, *p* < 0.0001**)	NR
Natarajan 2019 India (31)	DR	10	N	N	N	Sensitivity for detection of AI referable Diabetic Retinopathy (RDR) remained at 100% (95% CI, **78.2–100.0%**), while the specificity was 81.9% (95% CI, **75.9–87.0%**).	NR

DR, Diabetic retinopathy; C, Cataract; NR, Not reported.

An RCT in Rwanda investigated whether DR Screening using retinal imaging with AI interpretation delivered immediately to patients was superior to human-interpreted reports delivered several days later. It turned out, the photos and immediacy of results improved referral uptake within a month for rural residents, older patients and men, but not for women when compared to delayed SMS results and phone call advising of the need to visit the clinic (29). Competing cultural priorities such as caring, childminding roles, or restricted access to income could have hindered women’s opportunity to take up the specialist visit offered on the same day of the AI-supported results. A cross-sectional comparison of AI generated analysis of fundus looking for referable DR showed promising sensitivity and moderate specificity against ophthalmologist assessment.

### 3.4 Success factors for scalability and sustainability findings

Since studies were generally short-term and many had retrospective or cross-sectional designs, only six suggested the potential for longer-term maintenance but without demonstrable indicators (44, 47, 50, 53, 55, 56).

The multicomponent outreach in India which achieved high attendance rates suggested that a combination of free teleophthalmology consults with low-cost medication and treatment would contribute to sustaining the program past the project cycle (50). While the World Health Organization has called for continuity of care through integration of eye health into the routine health system functions (3), we found only one example of this strategy among the reports not assessable for quality (55). That report was a mixed methods study evaluating a 3-year initiative in Bangladesh where an education campaign prepared a redesign of eye health screening, diagnosis and management opportunities within diabetes routine services at the primary, secondary and tertiary levels. This required a re-structuring of the horizontal and vertical referral system which proved quantitatively that public awareness had succeeded, but the claimed potential sustainability and patient compliance with referral and treatment was based on qualitative self-reported data.

Three reports where risk of bias could not be assessed provided a qualitative perspective indicating that further health promotion training for rural ophthalmic staff, changed community knowledge and attitudes towards eye health through public education including the seriousness of diabetes complications, digital data tracking with text reminders to ensure appointment adherence, and transport or treatment subsidies were possible areas for sustainable intervention in Pakistan and Bangladesh (47, 53, 56). A before-after evaluation of the outreach program integrated within other district services in Timor-Leste considered the integration affordable within existing resources and appropriate to reach rural populations otherwise not able to access screening (44). Likewise, a low quality descriptive cross-sectional study assessing cataract surgical camps in India found the establishment of protocolised safe operating theatres near the target population affordable and suggested that adding education to address fear in patients with low literacy could enhance participation (45). Tangible measurements of sustainability from higher quality designs are required to confirm all these inferences. No study reported concrete insights into how eye care interventions could be integrated into diabetes or healthy ageing screening or management programs for sustainability.

### 3.5 Intervention components and providers to inform future services

In answering our third research question, broadly speaking, the components of interventions that worked to increase access and the providers delivering them are described below for the interventions tested in high and moderate quality studies. Specific details of all eligible interventions are in Supplementary Tables S5.1–S5.5 alongside the usual care descriptions when available.

#### 3.5.1 Teleophthalmology accuracy

Generally, these studies involved a trained remote technician, ophthalmic nurse or non-medical grader or a team of these assessing patients in a country-level facility with a smartphone camera in Brazil, China and Nepal (23, 30, 36) or PEEK retina software in Uganda (34) to capture fundus photograph or retinal video. These were transferred to a secondary or tertiary center for assessment of picture quality and subsequent diagnosis by an ophthalmologist. In India the remote assessment was conducted during real-time specialist consultation via telehealth and electronic medical records (20). In the cases of China, Nepal and India, technicians also provided initial diagnostic impression and referral for confirmation at the urban centre and inter-rater agreement for diagnostic accuracy was measured (20, 30, 36). The teleophthalmology program in Argentina was not described (39). Usual care was generally the in-person examination by a specialist or ophthalmologist in training at a large and higher-level ophthalmology or diabetes facility.

#### 3.5.2 Outreach camps

Described for China, India and Iran, it covered two main approaches: Screening and treatment camps consisting of multidisciplinary teams of ophthalmologist and ophthalmic nurses travelling outside main centers to reach vulnerable populations with or without the assistance of non-medical local health workers (32, 50); or screening led by non-medical community health workers based either at a rural primary care center, another temporary screening center or at people’s homes on a door-to-door fashion using a mobile application for subsequent referral to a higher level facility (35, 43, 49). Some also offered concurrent health education (50) or subsidized surgery (32).

#### 3.5.3 Health financing

The interventions in China (22, 32) consisted of total coverage of cataract surgery cost in secondary or tertiary hospitals, with additional transport reimbursement to enhance treatment attendance. The Vietnam study subsidized surgery in addition to either medical out-of-pocket expenses or other non-medical out-of-pocket expenses (24). And a post-operative study in China offered transport subsidy and phone reminder to increase follow-up attendance (25). Usual care comparators were low-cost surgery or full payment of medical out-of-pocket expenses.

#### 3.5.4 Public education

Three studies with very diverse educational components showed some overall effectiveness rather than specific differentials for our subgroups of interest. A Myanmar door-to-door education by community health educators once a month for 3 months led to improved access to eye healthcare (27). In Bangladesh, tertiary educated health workers delivered intensive and personalized education on the impact of non-compliance for 5 months and made telephone reminders before appointments for 3 months (26). By contrast, in China a 5-min video supplemented with 5-min counselling increased overall acceptance of cataract in rural areas (28). Usual care was generic information on treatment options and locations without personalized reminders.

#### 3.5.5 AI digital

Two studies used artificial intelligence generated DR grading reports to trigger uptake of referrals. In Rwanda, referral adherence was better for the group who received immediate AI report with promise of human cross checking days later than for the group that received the human grading report a month later and was unaware of AI reports (29). In India, the comparison of AI graded DR was satisfactory against human specialist grading (31). The China study used nurses to send mobile SMS reminders 1 week and 3 weeks before appointments and was superior to usual care of verbal reminder at the time of initial visit (21).

### 3.6 Sustainability as per RE-AIM framework

Our intention to map interventions to the RE-AIM framework to enable replication across contexts, assessment of generalizability, and to provide an implementation roadmap for readers, was limited by the eligible studies falling short on reporting several domains. The included studies generally described only two of the five RE-AIM dimensions: Reach of target population and Effectiveness. The Adoption, Implementation and Maintenance domains were rarely covered (44, 47, 50, 53, 55, 56) and only in a speculative fashion as mentioned in section 3.4, thus precluding determination of either: fidelity of delivery; participation rate; extent of representativeness; intervention time or cost; and maintenance or potential for sustainability -when reported- were often implied rather than measured through long-term follow-up. This was particularly true for control groups, mostly defined in trials or controlled cohorts. The lack of reporting of these implementation science domains has previously been identified, as well as the convenience in adaptation of the RE-AIM framework to suit the needs of different research projects across settings (15). Multiple factors may explain the lack of coverage of scalability and sustainability in the studies included in this review: the short duration of interventions without an evaluation or monitoring component; lack of follow-up due to limited time and/or funding; absence of governance structures to oversee long-term performance; lack of supportive policies to mandate continuity; and reduced local capacity or other resources to sustain the practice under investigation.

Findings from the studies with low quality scores of 6 and lower, and those not assessable due to lack of information again lacked focus on scalability or sustainability and can be seen in Supplementary Tables S6.1–S6.5.

## 4 Discussion

Findings from the 24 recent high-to-moderate quality interventions from 10 LMICs identified in this scoping review revealed mixed evidence of *potential* for effectiveness in reducing inequities. Results varied across health systems, based on heterogenous designs, and did not always provide direct outcome measures. In practice, unlike drug distribution for instance, each health system/country has different needs, culture, health literacy and dynamics where their influence on eye care effectiveness is challenging to measure and achieve. The absence of contextual information in effectiveness studies has been acknowledged before (57). Of the above 24 more credible studies, 16 reported actual effectiveness measures of inequity reduction for rural residents (21, 22, 25–27, 29, 32–35, 50), seven did so for women (23, 25, 32, 34, 35, 39, 50), and only three reported effective results specifically relevant to older people (27, 29, 35). The distribution of intervention types by target groups of the studies assessed for quality is summarized in Table 7.

**Table 7:** Synthesis of effectiveness by intervention type and target group (*n* = 33 studies).*

Study quality and intervention type	Effective for women	Effective for rural residents	Effective for older people
Moderate to high quality			
Teleophthalmology	R (23, 34, 39) P (30)	R (20, 23, 30, 36, 39) P (34)	P (30, 34)
Outreach camps	R (32, 35, 50)	R (32, 35, 43, 49, 50)	R (35)
Health financing	R (25) P (22, 24)	R (22, 25, 33)	–
Public education	P (27)	R (28)	R (27)
AI-digital	–	R (21, 29)	R (29)
Low quality			
Teleophthalmology	–	(37)	–
Outreach camps	R (45)	R (42, 45, 52)	R (42, 45, 52)
Health financing	–	–	–
Public education	–	–	–
AI-digital	N/A	N/A	N/A

(R) reported effectiveness or (P) presumed effectiveness as per methods section. –, not effective or effectiveness not reported (10 studies). N/A, not applicable (no studies found). *Results only for studies with quality assessment.

Results from nine studies had potential to reduce inequities but no sub-analysis for these groups were reported. Two studies (outreach and health education respectively) provided direct evidence of overall effectiveness where impact did not differ by gender (26, 49). And four studies provided indirect but measured evidence of benefit for women and/or rural residents via teleophthalmology accuracy (23, 30, 39) or health financing modeling (24). However, the eligible publications did not enable our answering of research question 4 on success factors for sustainability.

Two additional gaps identified by our search were the shortage of gender-specific interventions to reduce the access gap for women, and the limited number of studies targeting older people. These omissions underscore a broader gap in the literature on the mechanisms through which gendered and age inequities in eye care are produced and sustained. Both have implications for service policy as discussed below.

As highlighted by the Lancet Global Health Commission on Global Eye Health, achieving equity requires not only improving service availability but also addressing the social, cultural, and institutional conditions that constrain access for women and other underserved groups (1, 58). Gender-based disparities are often reinforced by structural factors such as caregiving burdens, limited control over financial resources, and restrictions on mobility and healthcare decision-making by the ways in which gender roles and norms are constructed and enforced (58). These systemic constraints often interact with other forms of exclusion related to age, disability, or rural residence, amplifying barriers to access (59). Additionally, women with vision loss may face heightened risks of economic insecurity, social isolation, and violence (60). To address these inequities, interventions must move beyond service delivery to transform the structural conditions that shape access. Promising approaches include women-led service models, community-based care, and gender-sensitive clinic adaptations such as separate queues for women and involving men as champions promoting eye care services for women and girls (61). Institutional change through sex-disaggregated data use, gender equity training for providers, and mentoring for women’s leadership in health systems has also been highlighted as critical for sustainable impact (62, 63).

The three studies that reported results for older people (27, 29, 35) covered the effectiveness of public education, AI retinal photography and outreach initiatives. These three broad types of interventions are often suitable for all adults (not exclusively older people). Strategies might need some tailoring for older populations to enable effectiveness testing because dedicating resources to only include older populations is likely to undermine scalability and sustainability. Based on prevalence studies and perspectives publications, such strategies for reducing older people’s inequitable eye care access could be embedded in the general healthcare access initiatives. Opportunities exist in primary care for coordination of identification and screening with associated referral to collaborating partners in other sectors (64). Yet, treatment success would not be possible without introducing or strengthening universal healthcare policies or health financing approaches to overcome age-related socioeconomic disadvantage that compounds multimorbidity and workforce disengagement (65, 66). This financial coverage could be coupled with safe service environments and followed by health risk surveillance to monitor the uptake of preventive behaviours (hygiene and screening in the case of eye care) as they are improved by a boost in socioeconomic circumstances. Interventions to enhance social participation and expand social support also have potential to improve older people’s access to care (66) by virtue of companionship networks to facilitate attendance to regular eye examinations and treatments.

Findings from outreach camps in Asia suggest that bringing screening closer to people in rural and remote areas does increase attendance by women and older people but most failed to report associated increases in treatment services utilization following diagnosis. This is an important gap in effectiveness studies worthy of further research. Outreach was more likely to be effective in home-based screening or initiatives in primary care supplemented with either health financing or an education campaign. A recent review investigating improved access to rural communities, confirmed that in light of local staff shortages, outreach, telehealth, partnering with NGOs and health financing schemes were essential ingredients for success (67). Outreach will likely continue to be a part of the eye care landscape but the challenges of community engagement and integration with other services to maintain sustainability warrant further investigation.

Teleophthalmology facilitates access to examination of people in remote areas. For about a decade, telehealth has been evaluated as a cost-effective measure if the screening interval is kept at 2–5 years (e.g., $1,320 per QUALY gained) (68). Our review found that in Asia and South America, training non-medical and non-optometrist technical staff in mobile telehealth equipment in rural camps linked to real-time advice by urban-based specialists enabled access to immediate diagnosis, decision-making and sometimes immediate transfer to treatment for women and people in rural and remote areas. However, inter-rater agreement and follow-up management were not always measured, hence overall effectiveness and sustainability cannot be confirmed. More comprehensive indicators of success across the patient care pathway need to be included in future studies.

In this review, health financing coverage for cataract surgery in Asia and Africa pointed in a promising direction particularly for rural residents, but improvements over time were not substantial despite some interventions also subsidizing transport. Possible explanations are that part subsidy of treatment still means considerable economic burden to patients in developing countries, given that out-of-pocket expenses also include loss income per visit and accommodation away from home (69) or that surgery acceptance might be higher for people with worse visual acuity and people with prior good post-operative outcome. While essential financial support might contribute to sustainability, cultural, psychosocial and attitudinal barriers (fear of surgery, distrust in the health system, pessimism) and other barriers beyond cost and transport could curb intervention success (70). These factors need to be investigated further to better understand the causes of only modest increases in surgical uptake and post-intervention follow-up found in this review.

A limited number of high-quality health education and counselling initiatives in Asia demonstrated improvements in adherence to DR referrals and slight increases in overall acceptance of cataract surgery. Higher intensity or frequency of personalized messages and reminders were more likely than standard once-off information to improve screening attendance, but subsequent referral compliance was similarly improved for women and men. Beyond knowledge improvements, other social determinants of referral uptake for women such as gender-sensitive care, gender roles, balance of power in households, gender-based discrimination and other traditions and expectations can interfere with programs achieving success and need to be planned for (71). None of the studies explicitly addressed these issues.

The overall effectiveness of low-cost digital SMS reminders in Asia to capture defaulters was significant but perhaps additional intervention components need to be explored to enhance appointment attendance by all and by women in particular. Smartphone photography holds promise on grading DR, but claims may be overstated as its sensitivity for diagnosis leaves room for improvement. Artificial intelligence for automated instant interpretation as adjuvants encourages referral compliance and was considered effective in India and Africa where ophthalmologists were not available for immediate interpretation. However, the specialist assessment of fundus photographs will be needed for confirmation of both accuracy and effectiveness in increasing treatment completion.

Essential features of a sustainable strategy include ability to be implemented within existing financial resources or modest investment; acceptability among existing staff by not unreasonably requiring extensive training or impact on current workload; and minimal burden to patients or the health system (72). The studies included in this review generally did not report on these aspects to enable an objective assessment of sustainability, but the sister manuscript on the synthesis of the qualitative studies currently underway by other FHF colleagues may shed some light on perceptions of potential sustainability. The cost-effectiveness of eye health treatments in general have been promoted in light of their impact on quality of life and disability adjusted life years (73). Service integration has also been heralded as a cost-effective key to sustainability in low-resource settings. However, the practicalities of workforce skills shortage, lack of trust in community mobilisers, cultural and political conditions hindering referrals, and requirements of compatibility of health information systems, shared infrastructure, ongoing funding from several sources, and procurement coordination explain why eye care integration and sustainability have not been broadly achieved (74).

To attain equitable access to healthcare as per the sustainable development goals, the six pillars of a strengthened health system in WHO’s view have been identified as: service delivery; health workforce; information; medical products, vaccines and technologies; financing; and leadership and governance (stewardship) (75). Only a handful of these priorities were partly covered by the included studies as interventions generally had a discrete objective. Scarce resources and varying political support for action may contribute to the gap in the eye care accessibility that disadvantaged groups continue to experience.

### 4.1 Strengths and limitations of the review

To the best of our knowledge, this scoping review is a first attempt at synthesizing potential strategies to specifically address inequities of eye care access by disadvantaged groups in low-middle-income settings at a large scale. It compiles findings from diverse interventions in several low-resource countries and focused on higher quality scores to highlight credibility of certain interventions and enhance confidence in decision making for replication or application in routine practice. It is not possible to estimate the impact of attrition on the effectiveness estimate but we call for caution on the three high quality studies who were affected by it (30, 32, 41). While many publications failed to describe the intervention in detail to enable replication by others, our supplementary file 5 contains what component descriptions were extractable for future reference, guided by the TIDIER Framework (17). Our manuscript reporting followed the PRISMA Scr checklist (Supplementary Table S7). However, two deviations from protocol were required. As service delivery workers, our time and staff resource limitations made us confine the searches to PubMed and the relevant gray literature, hence some other studies indexed by other databases may have been missed. Yet, we believe this synthesis covering the largest publication database with representation from four world regions is a good start and we did not want to delay its release to stimulate debate and action. The second deviation consisted in our inclusion of accuracy studies which did not have a focus on women or older people but whose main outcomes were about concordance in clinical assessment that could potentially benefit rural residents. We made this decision given the high potential for immediate applicability to addressing access inequities in rural areas and generally referred to these studies as potentially effective, unless the authors of those manuscripts declared the actual effectiveness for our target groups, in which case we referred to their finding as actual effectiveness.

### 4.2 Implications for practice and future research

It is encouraging to find clear evidence-based direction where the limited funding for interventions in LMICs can be invested to enhance access for women and rural residents, although more evidence is needed of impact on older subgroups. Routine eye care services can now be confident that outreach initiatives and SMS advance reminders increase attendance to screening, and health financing schemes extending to transport costs improve willingness to undergo cataract surgery and actual surgical and follow-up rates. It is also clear that education as a stand-alone strategy is ineffective in enhancing referral compliance. The ongoing training of remote non-medical staff in using teleophthalmology should be pursued given the promising accuracy results.

Our supplementary files describe in detail the interventions content and intensity to facilitate replication. Combining human, financial and technical resources for multicomponent interventions may reach more vulnerable populations.

Given that less than half of the eligible studies were considered of high or moderate quality, we invite more rigorous evaluation of cost-effectiveness, patient-reported outcomes, and protocolised feasibility of integration into existing services to enhance access for women and other vulnerable groups. Inclusion of a formative phase to understand the complexities of the setting, use of implementation frameworks, and pre-agreed standard measures such as government and community involvement past the pilot phase, equity and inclusion, quality of life impacts, economic viability, may be laborious and challenging but would enable accurate estimation of scalability and sustainability. Quantification of clinical and public health effectiveness should be supplemented by evaluation of value for money, qualitative consultations to address other contextual barriers on the care pathway. Finally, more transparent reporting of the RE-AIM domains and more comprehensive outcome reporting on gender differentials for older people and rural residents would assist health service planners in assessing feasibility of implementation to attain the sustainable development goal 3 (Good health and wellbeing for all) and SDG 4 (gender equality).

Taking advantage of the high level of attention that scalability of some interventions has received in recent times, uptake of those recommendations is overdue in low-income settings. For teleophthalmology to overcome barriers of remote care, scalability strategies might include not only capacity building of local health workers but also education and collaboration with clinicians to influence their acceptability, integration into other health services, expanded scope of telehealth to include other conditions, alignment of information systems, and partnership with government or public sector to ensure infrastructure funding (76). Expansion of coverage in outreach initiatives is thought to be achievable through multisector sustainable funding for centers to produce net revenue, addressing social aspects as transport subsidies to the sites, employing school graduates in screening centers, and appointing village health guardians for supplementary door-to-door screening and referral to prevent service underutilization (7). The World Health Organization has identified that “*System expansion does not always improve access”* and that the key is to customize demand and supply of financial incentives to the specific country and health system. Adaptable funding for programming in changing environments supported by evaluation of value for money has also been suggested by other non-profits in their advocacy to reach universal eye health coverage (77). Finally, scaling community education on the personal and social impact of blindness to increase public demand for services cannot work in isolation of affordability or local ownership by committed leaders (78).

### 4.3 Conclusion

This scoping review contributes to the understanding of the challenges and opportunities in addressing access inequities to eye health by groups that should not be left behind. Our findings show evidence that several strategies warrant consideration and investment to reduce gender and geographic inequities in eye care access: well-conducted outreach screening camps delivered by multidisciplinary groups; training of local non-medical graders for accurate preliminary diagnosis to be confirmed by specialists via teleophthalmology; health financing schemes that support both cataract surgery and transport costs. Multicomponent interventions may hold the clue to closing the inequities gap for vulnerable groups such as teleophthalmology plus and intensive ongoing health education to enhance referral compliance in areas of specialist shortages. In addition, the combination of SMS appointment reminders, preliminary AI-driven reporting of fundus photography could also be used as adjuvants for any of the above. These selected strategies signal a good starting point for replication and larger scale implementation after addressing the design and reporting weaknesses. Future research should prioritize cost-effectiveness analyses and long-term sustainability assessments, particularly for interventions targeting women, older people and rural residents.

## Footnotes

^1^ https://osf.io/yr7tg/?view_only=968ba9e8c910470ca227dcdb0da3cda8
