# Supplementary material for: Eye care interventions that reduce access inequities for women, rural residents and older people in low-middle-income countries: a scoping review
Source: Front Public Health. 2025 Jun 23;13:1578848. doi: 10.3389/fpubh.2025.1578848 (PMC12230066; doi:10.3389/fpubh.2025.1578848)
Supplement: Supplementary file 1 [file Data_Sheet_1.docx]

**Supplementary files 1 - 7**

| **Table #** | **Content** | **Page number** |
| --- | --- | --- |
| S1 | PubMed Search strategy | 1-3 |
| S2 | S2.1 Risk of bias assessment criteria and S2.2 study ranking | 3-6 |
| S3 | Detailed reasons for exclusion | 7-10 |
| S4 | Detailed study characteristics | 11-17 |
| S5 | Detailed intervention descriptions (Tables S5.1 to S5.5) | 18-32 |
| S6 | Detailed results for low quality and non-assessable studies (Tables 6.1-6.5) | 33-36 |
| S7 | PRISMA-Scr Checklist | 37-38 |

**S1. PubMed search strategy**

| **1** | (eye health[Tiab] OR vision[Tiab] OR “eye screening”[Tiab] OR “eye surgery”[Tiab] OR “eye hospital”[Tiab] OR cataract[Tiab] OR “diabetic retinopathy”[Tiab] OR trachoma[Tiab] OR ophthalmologist[Tiab] OR blind[Tiab] OR blindness[Tiab])  Limited to 2002 to 2023 139,845 |
| --- | --- |
| **2** | (Inequalities[Tiab] OR disparity[Tiab] OR disparities[Tiab] OR “health service access”[Tiab] OR remote[Tiab] OR rural[Tiab] OR disadvantaged[Tiab] OR vulnerable[Tiab] OR poor[Tiab] OR disable[Tiab] OR disabled[Tiab] OR disability[tiab] OR “visually impaired”[Tiab] OR Woman[Tiab] OR Women[Tiab] OR female[Tiab] OR girls[Tiab])  Limited to 2002 to 2023 555,676 |
| **3** | #1 AND #2 Limited to 2002 to 2023 22,256 |
| **4** | (afghanistan[Tiab] OR albania[Tiab] OR algeria[Tiab] OR american samoa[Tiab] OR angola[Tiab] OR antigua[Tiab] OR barbuda[Tiab] OR argentina[Tiab] OR armenia[Tiab] OR armenian[Tiab] OR aruba[Tiab] OR azerbaijan[Tiab] OR bahrain[Tiab] OR bangladesh[Tiab] OR barbados[Tiab] OR belarus[Tiab] OR byelarus[Tiab] OR belorussia[Tiab] OR byelorussian[Tiab] OR belize[Tiab] OR british honduras[Tiab] OR benin[Tiab] OR dahomey[Tiab] OR bhutan[Tiab] OR bolivia[Tiab] OR bosnia[Tiab] OR herzegovina[Tiab] OR botswana[Tiab] OR bechuanaland[Tiab] OR brazil[Tiab] OR brasil[Tiab] OR bulgaria[Tiab] OR burkina faso[Tiab] OR burkina fasso[Tiab] OR upper volta[Tiab] OR burundi[Tiab] OR urundi[Tiab] OR cabo verde[Tiab] OR cape verde[Tiab] OR cambodia[Tiab] OR kampuchea[Tiab] OR khmer republic[Tiab] OR cameroon[Tiab] OR cameron[Tiab] OR cameroun[Tiab] OR central african republic[Tiab] OR ubangi shari[Tiab] OR chad[Tiab] OR chile[Tiab] OR china[Tiab] OR colombia[Tiab] OR comoros[Tiab] OR comoro islands[Tiab] OR mayotte[Tiab] OR congo[Tiab] OR zaire[Tiab] OR costa rica[Tiab] OR cote d’ivoire[Tiab] OR cote d’ ivoire[Tiab] OR cote divoire[Tiab] OR cote d ivoire[Tiab] OR ivory coast[Tiab] OR croatia[Tiab] OR cuba[Tiab] OR cyprus[Tiab] OR czech republic[Tiab] OR czechoslovakia[Tiab] OR djibouti[Tiab] OR french somaliland[Tiab] OR dominica[Tiab] OR dominican republic[Tiab] OR ecuador[Tiab] OR egypt[Tiab] OR united arab republic[Tiab] OR el salvador[Tiab] OR equatorial guinea[Tiab] OR spanish guinea[Tiab] OR eritrea[Tiab] OR estonia[Tiab] OR eswatini[Tiab] OR swaziland[Tiab] OR ethiopia[Tiab] OR fiji[Tiab] OR gabon[Tiab] OR gabonese republic[Tiab] OR gambia[Tiab] OR georgia[Tiab] OR georgian[Tiab] OR ghana[Tiab] OR gold coast[Tiab] OR gibraltar[Tiab] OR greece[Tiab] OR grenada[Tiab] OR guam[Tiab] OR guatemala[Tiab] OR guinea[Tiab] OR guyana[Tiab] OR guiana[Tiab] OR haiti[Tiab] OR hispaniola[Tiab] OR honduras[Tiab] OR hungary[Tiab] OR india[Tiab] OR indonesia[Tiab] OR timor[Tiab] OR iran[Tiab] OR iraq[Tiab] OR isle of man[Tiab] OR jamaica[Tiab] OR jordan[Tiab] OR kazakhstan[Tiab] OR kazakh[Tiab] OR kenya[Tiab] OR korea[Tiab] OR kosovo[Tiab] OR kyrgyzstan[Tiab] OR kirghizia[Tiab] OR kirgizstan[Tiab] OR kyrgyz republic[Tiab] OR kirghiz[Tiab] OR laos[Tiab] OR lao pdr[Tiab] OR lao people's democratic republic[Tiab] OR latvia[Tiab] OR lebanon[Tiab] OR lesotho[Tiab] OR basutoland[Tiab] OR liberia[Tiab] OR libya[Tiab] OR libyan arab jamahiriya[Tiab] OR lithuania[Tiab] OR macau[Tiab] OR macao[Tiab] OR macedonia[Tiab] OR madagascar[Tiab] OR malagasy republic[Tiab] OR malawi[Tiab] OR nyasaland[Tiab] OR malaysia[Tiab] OR maldives[Tiab] OR indian ocean[Tiab] OR mali[Tiab] OR malta[Tiab] OR micronesia[Tiab] OR kiribati[Tiab] OR marshall islands[Tiab] OR nauru[Tiab] OR northern mariana islands[Tiab] OR palau[Tiab] OR tuvalu[Tiab] OR mauritania[Tiab] OR mauritius[Tiab] OR mexico[Tiab] OR moldova[Tiab] OR moldovian[Tiab] OR mongolia[Tiab] OR montenegro[Tiab] OR morocco[Tiab] OR ifni[Tiab] OR mozambique[Tiab] OR portuguese east africa[Tiab] OR myanmar[Tiab] OR burma[Tiab] OR namibia[Tiab] OR nepal[Tiab] OR netherlands antilles[Tiab] OR nicaragua[Tiab] OR niger[Tiab] OR nigeria[Tiab] OR oman[Tiab] OR muscat[Tiab] OR pakistan[Tiab] OR panama[Tiab] OR papua new guinea[Tiab] OR paraguay[Tiab] OR peru[Tiab] OR philippines[Tiab] OR philipines[Tiab] OR phillipines[Tiab] OR phillippines[Tiab] OR poland[Tiab] OR polish people's republic[Tiab] OR portugal[Tiab] OR portuguese republic[Tiab] OR puerto rico[Tiab] OR romania[Tiab] OR russia[Tiab] OR russian federation[Tiab] OR ussr[Tiab] OR soviet union[Tiab] OR union of soviet socialist republics[Tiab] OR rwanda[Tiab] OR ruanda[Tiab] OR samoa[Tiab] OR pacific islands[Tiab] OR polynesia[Tiab] OR samoan islands[Tiab] OR sao tome and principe[Tiab] OR saudi arabia[Tiab] OR senegal[Tiab] OR serbia[Tiab] OR seychelles[Tiab] OR sierra leone[Tiab] OR slovakia[Tiab] OR slovak republic[Tiab] OR slovenia[Tiab] OR melanesia[Tiab] OR solomon island[Tiab] OR solomon islands[Tiab] OR norfolk island[Tiab] OR somalia[Tiab] OR south africa[Tiab] OR south sudan[Tiab] OR sri lanka[Tiab] OR ceylon[Tiab] OR saint kitts and nevis[Tiab] OR st kitts and nevis[Tiab] OR saint lucia[Tiab] OR st lucia[Tiab] OR saint vincent[Tiab] OR st vincent[Tiab] OR grenadines[Tiab] OR sudan[Tiab] OR suriname[Tiab] OR surinam[Tiab] OR syria[Tiab] OR syrian arab republic[Tiab] OR tajikistan[Tiab] OR tadjikistan[Tiab] OR tadzhikistan[Tiab] OR tadzhik[Tiab] OR tanzania[Tiab] OR tanganyika[Tiab] OR thailand[Tiab] OR siam[Tiab] OR timor leste[Tiab] OR east timor[Tiab] OR togo[Tiab] OR togolese republic[Tiab] OR tonga[Tiab] OR trinidad[Tiab] OR tobago[Tiab] OR tunisia[Tiab] OR turkey[Tiab] OR turkmenistan[Tiab] OR turkmen[Tiab] OR uganda[Tiab] OR ukraine[Tiab] OR uruguay[Tiab] OR uzbekistan[Tiab] OR uzbek[Tiab] OR vanuatu[Tiab] OR new hebrides[Tiab] OR venezuela[Tiab] OR vietnam[Tiab] OR viet nam[Tiab] OR middle east[Tiab] OR west bank[Tiab] OR gaza[Tiab] OR palestine[Tiab] OR yemen[Tiab] OR yugoslavia[Tiab] OR zambia[Tiab] OR zimbabwe[Tiab] OR northern rhodesia[Tiab] OR global south[Tiab] OR africa south of the sahara[Tiab] OR sub saharan africa[Tiab] OR subsaharan africa[Tiab] OR central africa[Tiab] OR north africa[Tiab] OR northern africa[Tiab] OR magreb[Tiab] OR maghrib[Tiab] OR sahara[Tiab] OR southern africa[Tiab] OR east africa[Tiab] OR eastern africa[Tiab] OR west africa[Tiab] OR western africa[Tiab] OR west indies[Tiab] OR indian ocean islands[Tiab] OR caribbean[Tiab] OR central america[Tiab] OR latin america[Tiab] OR south america[Tiab] OR central asia[Tiab] OR north asia[Tiab] OR northern asia[Tiab] OR southeastern asia[Tiab] OR south eastern asia[Tiab] OR southeast asia[Tiab] OR south east asia[Tiab] OR western asia[Tiab] OR east europe[Tiab] OR eastern europe[Tiab] OR developing country[Tiab] OR developing countries[Tiab] OR developing nation[Tiab] OR developing nations[Tiab] OR developing population[Tiab] OR developing populations[Tiab] OR developing world[Tiab] OR less developed country[Tiab] OR less developed countries[Tiab] OR less developed nation[Tiab] OR less developed nations[Tiab] OR less developed world[Tiab] OR lesser developed countries[Tiab] OR lesser developed nations[Tiab] OR under developed country[Tiab] OR under developed countries[Tiab] OR under developed nations[Tiab] OR under developed world[Tiab] OR underdeveloped country[Tiab] OR underdeveloped countries[Tiab] OR underdeveloped nation[Tiab] OR underdeveloped nations[Tiab] OR underdeveloped population[Tiab] OR underdeveloped populations[Tiab] OR underdeveloped world[Tiab] OR middle income country[Tiab] OR middle income countries[Tiab] OR middle income nation[Tiab] OR middle income nations[Tiab] OR middle income population[Tiab] OR middle income populations[Tiab] OR low income country[Tiab] OR low income countries[Tiab] OR low income nation[Tiab] OR low income nations[Tiab] OR low income population[Tiab] OR low income populations[Tiab] OR lower income country[Tiab] OR lower income countries[Tiab] OR lower income nations[Tiab] OR lower income population[Tiab] OR lower income populations[Tiab] OR underserved countries[Tiab] OR underserved nations[Tiab] OR underserved population[Tiab] OR underserved populations[Tiab] OR under served population[Tiab] OR under served populations[Tiab] OR deprived countries[Tiab] OR deprived population[Tiab] OR deprived populations[Tiab] OR poor country[Tiab] OR poor countries[Tiab] OR poor nation[Tiab] OR poor nations[Tiab] OR poor population[Tiab] OR poor populations[Tiab] OR poor world[Tiab] OR poorer countries[Tiab] OR poorer nations[Tiab] OR poorer population[Tiab] OR poorer populations[Tiab] OR developing economy[Tiab] OR developing economies[Tiab] OR less developed economy[Tiab] OR less developed economies[Tiab] OR underdeveloped economies[Tiab] OR middle income economy[Tiab] OR middle income economies[Tiab] OR low income economy[Tiab] OR low income economies[Tiab] OR lower income economies[Tiab] OR low gdp[Tiab] OR low gnp[Tiab] OR low gross domestic[Tiab] OR low gross national[Tiab] OR lower gdp[Tiab] OR lower gross domestic[Tiab] OR lmic[Tiab] OR lmics[Tiab] OR third world[Tiab] OR lami country[Tiab] OR lami countries[Tiab] OR transitional country[Tiab] OR transitional countries[Tiab] OR emerging economies[Tiab] OR emerging nation[Tiab] OR emerging nations[Tiab])  Limited to 2002 to 2023 212,695 |
| **5** | #3 and #4 Limited to 2002 to 2023 2,451 |
| **6** | (Effective*[tiab] OR intervention[tiab] OR solution[tiab] OR strateg*[tiab] OR impact[tiab] OR alert*[tiab] OR reminder[tiab] OR education*[tiab] OR mHealth[tiab] OR mobile[tiab] OR decision support*[tiab] OR  outreach[tiab]  OR camp[tiab] OR artificial intelligence*[tiab] OR  telehealth[tiab]  OR telehealth[tiab] OR telemedicine[tiab] OR teleophthalmology[tiab] OR behavioral intervention*[tiab] OR public campaign[tiab] OR  health system integration[tiab] OR health financing[tiab] OR universal coverage*[tiab])  Limited to 2002 to 2023 1,377,365 |
| **7** | #5 and #6 Limited to 2002 to 2023 1,357 |

**S2.1 Risk of bias assessment criteria**

| Below are the risk of bias questions for all eligible peer-reviewed studies (reports excluded):   1. Were there clear inclusion and exclusion criteria? 2. Was the sampling frame a true or close representation of the target population? 3. Was some form of random selection used to select the sample, OR was a full census undertaken? 4. Was the likelihood of nonresponse/attrition bias/missing data minimal (<20%)? 5. Was an acceptable case definition used in the study? 6. Was there some form of validation of the study instrument or definition that measured the parameter of interest 7. Were confounding factors considered, documented, and taken into account in the analysis? 8. Were the numerator(s) and denominator(s) for the parameter of interest appropriate? 9. Can we be confident that exposure data extraction was objective, standard for all subjects and accurate? 10. Did analysis follow pre-specified analysis plan? 11. Was there selective outcome reporting? 12. Were conclusions supported by the findings? |
| --- |

**S 2.2 Distribution of risk of bias of included studies (n=39)**

|  | **Number of studies** | | |
| --- | --- | --- | --- |
| **Quality criteria** | **Low** | **Medium** | **High** |
| Were there clear inclusion and exclusion criteria? | 31 | 6 | 2 |
| Was **the sampling frame** a true or close representation of the target population? | 24 | 11 | 4 |
| Was some form of random selection used to select the sample, OR was a full census taken? | 25 | 5 | 9 |
| Was the likelihood of non-response/attrition bias/missing data minimal (<20%)? | 22 | 5 | 12 |
| Was an acceptable case definition used in the study? | 34 | 5 | 0 |
| Was there some form of validation of the study instrument or definition that measured the parameter of interest? | 28 | 11 | 0 |
| Were confounding factors considered, documented, and taken into account in the analysis? | 21 | 7 | 11 |
| Were the numerator(s) and denominator(s) for the parameter of interest appropriate? | 30 | 4 | 5 |
| Can we be confident that exposure data extraction was objective, standard for all subjects and accurate? | 22 | 10 | 7 |
| Did analysis follow pre-specified analysis plan? | 32 | 5 | 2 |
| Was there selective outcome reporting? | 30 | 6 | 3 |
| Were conclusions supported by the findings? | 33 | 5 | 1 |

**S2.3 Diagram representing individual study quality ranking**

| Author | Year | Were there clear inclusion and exclusion criteria? | Was the sampling frame a true or close representation of the target population? | Was some form of random selection used to select the sample, OR was a full census taken? | Was the likelihood of non-response/attrition bias/missing data minimal (<20%)? | Was an acceptable case definition used in the study? | Was there some form of validation of the study instrument or definition that measured the parameter of interest? | Were confounding factors considered, documented, and taken into account in the analysis? | Were the numerator(s) and denominator(s) for the parameter of interest appropriate? | Can we be confident that exposure data extraction was objective, standard for all subjects and accurate? | Did analysis follow pre-specified analysis plan? | Was there selective outcome reporting? | Were conclusions supported by the findings? | Score out of 12 |
| --- | --- | --- | --- | --- | --- | --- | --- | --- | --- | --- | --- | --- | --- | --- |
| Khair | 2020 |  |  |  |  |  |  |  |  |  |  |  |  | 12 |
| Chen | 2018 |  |  |  |  |  |  |  |  |  |  |  |  | 11 |
| Essue | 2020 |  |  |  |  |  |  |  |  |  |  |  |  | 11 |
| Zhang | 2013 |  |  |  |  |  |  |  |  |  |  |  |  | 11 |
| Bobb-Semple | 2017 |  |  |  |  |  |  |  |  |  |  |  |  | 10 |
| Mathenge | 2022 |  |  |  |  |  |  |  |  |  |  |  |  | 10 |
| Natarajan | 2019 |  |  |  |  |  |  |  |  |  |  |  |  | 10 |
| Chen | 2011 |  |  |  |  |  |  |  |  |  |  |  |  | 10 |
| Ko | 2021 |  |  |  |  |  |  |  |  |  |  |  |  | 10 |
| de Araujo | 2021 |  |  |  |  |  |  |  |  |  |  |  |  | 10 |
| McKenna | 2018 |  |  |  |  |  |  |  |  |  |  |  |  | 9 |
| Zhang | 2010 |  |  |  |  |  |  |  |  |  |  |  |  | 9 |
| Huang | 2012 |  |  |  |  |  |  |  |  |  |  |  |  | 9 |
| Liu | 2012 |  |  |  |  |  |  |  |  |  |  |  |  | 9 |
| Xiao | 2022 |  |  |  |  |  |  |  |  |  |  |  |  | 8 |
| Yusuf | 2022 |  |  |  |  |  |  |  |  |  |  |  |  | 8 |
| Das | 2019 |  |  |  |  |  |  |  |  |  |  |  |  | 7 |
| Queiroz | 2020 |  |  |  |  |  |  |  |  |  |  |  |  | 7 |
| Mohan | 2012 |  |  |  |  |  |  |  |  |  |  |  |  | 7 |
| Ramagiri | 2020 |  |  |  |  |  |  |  |  |  |  |  |  | 7 |
| Collon | 2020 |  |  |  |  |  |  |  |  |  |  |  |  | 7 |
| Katibeh | 2020 |  |  |  |  |  |  |  |  |  |  |  |  | 7 |
| Amritanand | 2018 |  |  |  |  |  |  |  |  |  |  |  |  | 7 |
| Ortiz-Basso | 2019 |  |  |  |  |  |  |  |  |  |  |  |  | 7 |
| Kessy | 2007 |  |  |  |  |  |  |  |  |  |  |  |  | 6 |
| Raman | 2011 |  |  |  |  |  |  |  |  |  |  |  |  | 6 |
| Chariwala | 2020 |  |  |  |  |  |  |  |  |  |  |  |  | 6 |
| Murthy | 2020 |  |  |  |  |  |  |  |  |  |  |  |  | 5 |
| Okoye | 2015 |  |  |  |  |  |  |  |  |  |  |  |  | 5 |
| Adepoju | 2022 |  |  |  |  |  |  |  |  |  |  |  |  | 5 |
| Keshvadoorst | 2020 |  |  |  |  |  |  |  |  |  |  |  |  | 4 |
| Sangameswaran | 2016 |  |  |  |  |  |  |  |  |  |  |  |  | 4 |
| John | 2015 |  |  |  |  |  |  |  |  |  |  |  |  | 1 |

**S3. Detailed reasons for exclusion**

| serial # | **Full reference** | **Pre-coded reason** |
| --- | --- | --- |
| 1 | John S, Srinivasan S, Ram K et al 2022. Efficacy of an Automated Algorithm for Screening Diabetic Retinopathy in Gradable and Ungradable Images in Real-Time Conditions. Telemedicine and e-health 2022;29 (6): DOI 10.1089/tmj.2022.0113 | Ineligible study design |
| 2 | Rachapelle, S., et al., The cost-utility of telemedicine to screen for diabetic retinopathy in India. Ophthalmology, 2013. 120(3): p. 566-573. | Ineligible study design |
| 3 | Alam F, Chongsuvivatwong V, Mahmud H, Gupta PS (2013). Comparison of accessibility among vision-impaired patients visiting mobile and stationary hospitals in rural Bangladesh. HEALTH POPUL NUTR 2013 Jun;31(2):223-230 | Ineligible study design |
| 4 | Huang J, Du K, Guan H, Ding Y, Zhang Y, Wang D, et al. The Role of Village Doctors in Residents' Uptake of Eye Screening: Evidence from Ageing Residents in Rural China. Healthcare (Basel). **2022**;10(7). | Ineligible study design |
| 5 | Sightsavers Research CentreDepartment of International development., Cataract Surgery Uptake in Uganda. Final report 2018. https://research.sightsavers.org/wp-content/uploads/2018/05/Cataract-Surgery-Uptake-in-Uganda-final-report.pdf | Ineligible study design |
| 6 | Sightsavers Research Centre, Cataract Surgery Uptake in Zambia. 2020. https://research.sightsavers.org/project/cataract-surgery-uptake-in-zambia/ | Ineligible study design |
| 7 | Jafferji, SS. Screening for diabetic retinopathy: an overview approach for the Eastern African Region. JOECSA. 2012; 16(1) | Ineligible study design |
| 8 | Tilahuna and Fenta 2018; Coverage of azithromycin mass treatment for trachoma elimination in Northwestern Ethiopia: a community based cross-sectional study. BMC Ophthalmology (2018) 18:193  https://doi.org/10.1186/s12886-018-0868-1 | No effectiveness intervention |
| 9 | Courtright P, Murenzi J, Mathenge W, Munana J, Müller A. Reaching rural Africans with eye care services: findings from primary eye care approaches in Rubavu District, Rwanda. Trop Med Int Health. 2010 Jun;15(6):692-6. doi: 10.1111/j.1365-3156.2010.02530.x. Epub 2010 Mar 29. PMID: 20374559 | No effectiveness intervention |
| 10 | Baruwa E, Tzu J, Congdon N, He M, Frick KD. Reversal in gender valuations of cataract surgery after the implementation of free screening and low-priced high-quality surgery in a rural population of southern China. Ophthalmic Epidemiol. **2008**;15(2):99-104. | No effectiveness intervention |
| 11 | Fred Hollows Foundation, Financial barriers to and hardship from accessing services for cataract in Yunnan Province, China. 2018 | No effectiveness intervention |
| 12 | Sightsavers Tanzania, Gender Issues in Maono Project Areas. 2020 | No effectiveness intervention |
| 13 | Sharma M, Chakrabarty AS, Pavan R, et al. An integrated, mobile service for diabetic retinopathy in rural India. Community Eye Health 2011;24(75):17-8. | No effectiveness outcome of interest |
| 14 | Kuper H et al. 2010 Does cataract surgery alleviate poverty? Evidence from a multi-centre intervention study conducted in Kenya, the Philippines and Bangladesh. PLOS One 2010;(5)11:e15431 | No effectiveness outcome of interest |
| 15 | Korn Melrbi FK, Barreto Melo G. Feasibility of screening for diabetic retinopathy using artificial intelligence, Brazil. Bull World Health Organ **2022**;100:643–647 | No effectiveness outcome of interest |
| 16 | Raj P et al., Diabetic retinopathy screening uptake after health education with or without retinal imaging within the facility in two AYUSH hospitals in Hyderabad, India: A nonrandomized pilot study. Indian J Ophthalmol **2020** Vol. 68 Issue Suppl 1 Pages S56-s58 | No effectiveness outcome of interest |
| 17 | Fred Hollows Foundation, Pacific Diabetic Retinopathy Report. 2018 (amended 2019) | No effectiveness outcome of interest |
| 18 | Proser M, Shin P. The role of community health centers in responding to disparities in visual health. Optometry 2008;79(10):564-75. doi: 10.1016/j.optm.2008.04.101 | No LMI country |
| 19 | Mafwiri et al 2016; Mixed methods evaluation of a primary eye care training programme for primary health workers in Morogoro Tanzania. BMC Nursing (2016) 15:41 DOI 10.1186/s12912-016-0163-5 | No target eye condition |
| 20 | Rahman, M. J., Rahman, M. M., Matsuyama, R., Tsunematsu, M., Islam, R., Ahmed, A., ... & Kakehashi, M. (2022). Feasibility and acceptability of telepathology system among the rural communities of Bangladesh: A pilot study. Journal of Family Medicine and Primary Care, 11(6), 2613-2619. | No target eye condition |
| 21 | Hall C, Anthony B. Hall, Joyse Mallya, Paul Courtright and Gerjo KokEstablishing a screening programme for diabetic retinopathy in Kilimanjaro Region, Tanzania using intervention mapping | No women or rural areas |
| 22 | HuangXM,, Bo‑Fan Yang, Wen‑Lin Zheng, Qun Liu, Fan Xiao, Pei‑Wen Ouyang, Mei‑Jun Li, Xiu‑Yun Li, Jing Meng, Tian‑Tian Zhang, Yu‑Hong Cui and Hong‑Wei Pan Cost-efectiveness of artifcial intelligence screening for diabetic retinopathy in rural China Huang et al. BMC Health Services Research (2022) 22:260 https://doi.org/10.1186/s12913-022-07655-6 | No women or rural areas |
| 23 | Almatas consulting, Piloting the treatment of retinopathy in India - DR and ROP | No women or rural areas |
| 24 | Gichuhi S et al. Evaluation of the Kenyatta National Hospital diabetic retinopathy screening program 2015-2016. JOECSA. 2017; 21(2): 40-44 | No women or rural areas |
| 25 | Finger, R. P., Ali, M., Earnest, J., & Nirmalan, P. K. (**2007**). Cataract surgery in Andhra Pradesh state, India: an investigation into uptake following outreach screening camps. Ophthalmic epidemiology, 14(6), 327-332. | Qualitative study |
| 26 | Aboe A, Joof BM, Kanyi SK, Hydara A, Downs P, Bush S, Courtright P. The Gambia has eliminated trachoma as a public health problem: Challenges and successes. PLoS Negl Trop Dis. 2022 Mar 28;16(3):e0010282. doi: 10.1371/journal.pntd.0010282. PMID: 35344553; PMCID: PMC8989289. | Qualitative study without relevant outcomes |
| 27 | **Adepoju FG,** Olokoba BL, Olatunji VA, et al. Community Eye Care Outreaches through Collaborations with Community-Based Organisations in Resource-Poor Settings in Ilorin, Nigeria. J West Afr Coll Surg **2022**;12(3):79-83. doi: 10.4103/jwas.jwas_151_22 [published Online First: 20221006] | Review |
| 28 | Murthy KR, Murthy PR, Kapur A, Owens DR. Mobile diabetes eye care: experience in developing countries. Diabetes Res Clin Pract. 2012;97(3):343-9. | Review |
| 29 | RamkeJ, PetkovicJ, WelchV, BlignaultI, GilbertC, BlanchetK, ChristensenR, ZwiAB, TugwellP. Interventions to improve access to cataract surgical services and their impact on equity in low- and middle-income countries. Cochrane Database of Systematic Reviews 2017, Issue 11. Art. No.: CD011307. DOI: 10.1002/14651858.CD011307.pub2. | Review |
| 30 | Blanchet K, Gordon I, Gilbert CE, Wormald R, Awan H. How to achieve universal coverage of cataract surgical services in developing countries: lessons from systematic reviews of other services. Ophthalmic epidemiology. 2012 Dec 1;19(6):329-39. | Review |
| 31 | Johnson MRD et al. A review of evidence of evaluate effectiveness of intervention strategies to address inequalities in eye health care. RNIB Report: RNIB/CEP/01. 2011 | Review |
| 32 | Mailu EW, Virendrakumar B, Bechange S, Jolley E, SchmidtE. Factors associated with the uptake of cataract surgery and interventions to improve uptake in low-and middle-income countries: A systematic review. PLoS ONE 2020 15(7): e0235699.https://doi.org/10.1371/journal. pone.0235699 | Review |
| 33 | Lee L, Moo E, Angelopoulos T, Yashadhana A. Integrated people-centered eye care: A scoping review on engaging communities in eye care in low-and middle-income settings. PloS one. 2023 Jan 19;18(1):e0278969. | Review |
| 34 | Malik M, Strang N, Campbell P, Jonuscheit S. Exploring eye care pathways, patient priorities and economics in Pakistan: A scoping review and expert consultation study with thematic analysis. Ophthalmic and Physiological Optics. 2022 Jul;42(4):694-716. | Review |
| 35 | Prathiba, V., & Rema, M. (2011). Teleophthalmology: a model for eye care delivery in rural and underserved areas of India. International journal of family medicine, 2011. | Review |

**S4. Detailed study characteristics by eligible study design, solution focus, resources and target condition (n=39)**

| *Study ID & country* | Setting | | |  |  |  |  | Target group(s) | Solution focus | | | | | | | | | Resources | | | | | | Target condition | | |
| --- | --- | --- | --- | --- | --- | --- | --- | --- | --- | --- | --- | --- | --- | --- | --- | --- | --- | --- | --- | --- | --- | --- | --- | --- | --- | --- |
|  | Tertiary hospital | District/other hospital | Primary Care / Community | Study design | # participants | % women | % rural |  | Public education | Patient education | Provider training | Practice guidelines | Service Incentives | Financial support | Technology | Outreach | Redesigning spaces | Existing personnel | New staff | | New equipment | Remote experts | Diabetic retinopathy | | Cataract | General Ophthalm |
| Matheneg, 2022  Rwanda | ✔ | ✔ |  | RCT | 823 screened; 275 in trial | 58.2 | 40.4 | Individuals aged ≥18 years with known DM and referable eye condition |  |  |  |  |  |  | ✔ |  |  | ✔ | | ✔ | ✔ |  | ✔ | |  |  |
| Chen, 2018  China |  | ✔ |  | RCT | 233 | 51.6 | 100 | Patients with DM |  | ✔ |  |  |  |  | ✔ |  |  | N/R | | N/R | ✔ |  | ✔ | |  |  |
| Khair, 2020  Bangladesh | ✔ |  | ✔ | RCT | 299 | 51.5 | N/R | Patients aged ≥18 years with T2DM |  | ✔ |  |  |  |  |  |  |  | ✔ | |  |  |  | ✔ | |  |  |
| Liu, 2012  China |  | ✔ |  | RCT | 434 | 57.4 | 100 | Patients aged ≥50 years with operable cataract |  | ✔ | ✔ | ✔ |  |  |  |  |  | ✔ | |  |  |  |  | | ✔ |  |
| Zhang, 2013, China |  | ✔ |  | Cluster RCT | 432 | 60.6 | N/R | Symptomatic patients aged ≥50 years with operable cataract |  | ✔ | ✔ | ✔ | ✔ | ✔ |  |  |  | ✔ | | ✔ |  |  |  | | ✔ |  |
| Katibeh, 2020  Iran |  |  | ✔ | Cluster RCT | 3312 | 54.6 | 51.3 | Individuals aged ≥50 years |  |  | ✔ |  |  |  | ✔ | ✔ |  | ✔ | |  | ✔ | ✔ |  | |  | ✔ |
| Queiroz 2020  Brazil |  |  | ✔ | Pro | 627 | 63.2 | N/R | Individuals aged ≥18 years with T2DM |  |  | ✔ |  |  |  | ✔ |  |  | ✔ | |  | ✔ | ✔ | ✔ | |  |  |
| Khan, 2022  Pakistan | ✔ | ✔ | ✔ | Pro | 393,759 | 66.9-81.9 | N/R | Entire population |  | ✔ | ✔ |  |  |  | ✔ | ✔ |  | ✔ | | ✔ | ✔ |  |  | | ✔ | ✔ |
| Mohan, 2012  India |  |  | ✔ | Pro | 23,380 | 55.8 | 100 | General population and people with DM | ✔ | ✔ | ✔ |  |  |  | ✔ | ✔ |  | N/R | | N/R | ✔ | ✔ | ✔ | |  |  |
| Murthy, 2020  India |  | ✔ |  | Pro | 64,455 | N/R | N/R | People with DM |  |  | ✔ |  |  |  | ✔ |  |  | ✔ | | N/R | ✔ |  | ✔ | |  |  |
| Awan, 2019  Pakistan | ✔ |  | ✔ | Pro | 49,939 | N/R | 100 | Individuals with DM or at risk of DM | ✔ |  | ✔ |  |  |  |  | ✔ |  | ✔ | |  |  |  | ✔ | |  |  |
| Kessy, 2007  Tanzania | ✔ |  | ✔ | Pro | 198 | N/R | N/R | Patients who previously declined cataract surgery |  | ✔ |  |  |  | ✔ |  |  |  | ✔ | |  |  |  |  | | ✔ |  |
| John, 2015  India | ✔ |  | ✔ | Pro | 19,634 | 48.7 | 100 | Individual requiring eye care |  | ✔ |  |  |  | ✔ | ✔ | ✔ |  | ✔ | | ✔ | ✔ | ✔ | ✔ | | ✔ | ✔ |
| ResInt, 2019/20  Bangladesh | ✔ |  | ✔ | Pro | 375,088 individuals  41,207 students | 58%  52.6% | N/R | Individuals requiring eye care |  |  | ✔ | ✔ |  |  |  | ✔ |  | ✔ | |  |  |  |  | | ✔ |  |
| WHO, 2023  Malaysia | ✔ |  | ✔ | Pro | 93,053 | NR | N/R | Individuals requiring cataract surgery |  |  |  |  |  |  |  | ✔ | ✔ | ✔ | | ✔ | ✔ |  |  | | ✔ |  |
| Adepoju*,* 2022  Nigeria |  |  | ✔ | Retr | 13,661 | 47.0 | 100 | Entire population |  |  |  |  |  | ✔ |  | ✔ |  | N/R | | N/R | ✔ |  |  | | ✔ | ✔ |
| Das, 2019  India | ✔ |  | ✔ | Retr | 15,001 | 37.7 | 100 | Patients seeking eye care |  |  |  |  |  |  | ✔ |  |  | ✔ | |  | ✔ | ✔ |  | | ✔ | ✔ |
| Okoye, 2015  Nigeria | ✔ |  |  | Retr | 376 | 42.3 | N/R | Cataract surgical patients |  |  |  |  |  | ✔ |  |  |  | ✔ | |  |  |  |  | | ✔ |  |
| Chen, 2011  China | ✔ | ✔ |  | Retr | 13,262 | N/R | N/R | Individuals undergone cataract surgery |  |  |  |  |  | ✔ |  |  |  | ✔ | |  |  |  |  | | ✔ |  |
| McKenna, 2018  China |  | ✔ |  | Retr | 375 | 52.3 | 100 | Patients aged ≥18 years with DM |  |  | ✔ |  |  |  | ✔ |  |  | ✔ | |  |  | ✔ | ✔ | |  |  |
| Amritanand, 2018  India |  | ✔ | ✔ | Retr | 18,534 | 49.6 | 72.9 | Individuals requiring eye care |  |  | ✔ |  |  |  |  | ✔ |  | ✔ | | ✔ |  |  |  | | ✔ | ✔ |
| Ortiz, 2022  Argentina |  |  | ✔ | B-A | 137 | 70.0 | 100 | People with DM in rural area |  |  |  |  |  |  | ✔ | ✔ |  |  | |  | ✔ |  | ✔ | |  |  |
| Ko, 2021  Myanmar |  |  | ✔ | B-A | 112 | 58.6 | 100 | Patients aged ≥50 years with cataracts |  | ✔ |  |  |  |  |  | ✔ |  |  | | ✔ |  |  |  | | ✔ |  |
| Pereira, 2012  Timor-Leste |  | ✔ | ✔ | B-A | 1991 | 45.0 | 100 | Individuals requiring eye care in remote location | ✔ |  |  |  |  |  |  | ✔ |  | ✔ | | ✔ | ✔ |  |  | | ✔ |  |
| Collon, 2020  Nepal |  |  | ✔ | B-A | 163 | 56 | 100 | Individuals aged ≥18 years with visual acuity ≤ 6/18 |  |  |  |  |  |  |  | ✔ |  | ✔ | |  | ✔ | ✔ |  | | ✔ | ✔ |
| Natarajan, 2019  India |  |  | ✔ | C-S | 213 | 51.6 | N/R | Patients with DM in urban capital city |  |  | ✔ |  |  |  | ✔ |  |  | N/R | | N/R | ✔ |  | ✔ | |  |  |
| Ramagiri, 2020  India |  |  | ✔ | C-S | 267 | 56.9 | 0 | Individuals with DM in urban slums |  | ✔ |  |  |  |  |  | ✔ |  |  | | ✔ |  |  | ✔ | |  |  |
| Raman, 2011  India |  | ✔ | ✔ | C-S | 511 | 35.1 | 100 | People with DM in rural areas |  |  |  |  |  |  | ✔ | ✔ |  | N/R | | N/R | ✔ | ✔ | ✔ | |  |  |
| Sangameswaran, 2016  India |  |  | ✔ | C-S | 2,021 | 53.6 | 100 | Patients requiring cataract surgery |  |  |  |  |  |  |  | ✔ |  | N/R | | N/R | ✔ |  |  | | ✔ |  |
| Yusuf, 2022  Uganda | ✔ |  |  | C-S | 286 | 74.5 | 0 | Patients aged ≥18 years with DM |  |  |  |  |  |  | ✔ |  |  | ✔ | |  | ✔ | ✔ | ✔ | |  |  |
| Zhang, 2010  China |  |  | ✔ | C-S | 1,040 | 70.1 | 100 | Individuals aged ≥40 years with vision impairment in at least one eye thought to be due to cataract |  |  |  |  |  |  |  | ✔ |  | ✔ | |  |  |  |  | | ✔ |  |
| Xiao, 2022  China |  | ✔ | ✔ | C-S | 579 | 59.5 | 100 | Individuals aged ≥65 years with DM |  |  |  |  |  |  |  | ✔ |  | ✔ | |  |  |  | ✔ | |  |  |
| Keshvardoost, 2020  Iran | ✔ |  |  | C-S | 125 | 37.5 | 0 | Patients with diabetes |  |  | ✔ |  |  |  | ✔ |  |  | ✔ | | ✔ | ✔ | ✔ | ✔ | |  |  |
| de Araujo, 2021  Brazil |  | ✔ |  | C-S | 118 | N/R | 0 | Patients with eye complaints |  |  | ✔ |  |  |  | ✔ |  |  | ✔ | |  |  | ✔ | ✔ | | ✔ |  |
| Chariwala, 2020  India |  | ✔ | ✔ | C-S | 300 | 50.6 | 100 | People aged >15 years with DM | ✔ | ✔ | ✔ |  | ✔ |  |  | ✔ |  | ✔ | |  | ✔ |  | ✔ | |  |  |
| Huang, 2012  China |  | ✔ |  | C-S | 426 | 65 | 100 | Patients undergone cataract surgery (>3 months post-op) |  |  |  |  |  | ✔ |  |  |  |  | | ✔ |  |  |  | | ✔ |  |
| ResInt, 2019  Bangladesh | ✔ | ✔ | ✔ | C-S | 228 | 58.0 | 100 | Individuals with DM | ✔ | ✔ |  |  |  |  |  | ✔ |  | N/R | | N/R |  |  | ✔ | |  |  |
| Bobb-Semple, 2017 Uganda | ✔ |  |  | C-S | 207 | 69.5 | NR | Patients aged ≥18 years with DM |  |  |  |  |  |  | ✔ |  |  | ✔ | |  | ✔ |  | ✔ | |  |  |
| Essue 2020, Viet Nam |  |  | NA | Mod | 20,051,000 | N/R | N/R | Patients aged ≥50 years with vision impairment due to cataract |  |  |  |  |  | ✔ |  |  |  |  | |  |  |  |  | | ✔ |  |

*B-A= Before-After design C-S Cross-sectional DM= diabetes mellitus Mod=Modelling N/R=not reported*

*Pro=prospective cohort RCT= randomized controlled/community trial Retr= retrospective analysis*

**S5. Description of activities for both intervention and comparison groups (greyed out are low quality studies and those not assessable for quality [N/A])**

**Outreach Interventions description by quality score** **(n=15)**

**S.5.1 OUTREACH INTERVENTIONS** description by quality score (n=15)

| **Author, publication year, country, quality score out of 12** | **Provider and setting**  **(who/where)** | **Intervention content/method dose/frequency**  **(what, how, how often, how long)** | **Usual care provider and**  **setting (who/where)** | **Comparator/Usual care dose/frequency** |
| --- | --- | --- | --- | --- |
| Zhang 2010, China  Quality: 9 | Who:Ophthalmologists, ophthalmic nurse  Where: Village camps of Jiazi Township | Outreach cataract screening programme offering low-cost cataract surgery.  Once off opportunity with pre-documented barriers for attendance and willingness to pay | Who: Ophthalmologist  Where: Eye clinic in Jiazi Township | Presenting at the local eye clinic for an eye examination, no offer of low-cost surgery |
| Xiao 2022, China  Quality: 8 | Who: non-medical graders  Where: Primary Health Centres | Group 2: Outreach screening cohort at the primary care centres  (no further description) | Group 1: Provider (Who and Where):  Trained local ophthalmologists for passive case finding at Secondary -level hospitals  Group 3: Provider (Who and Where): Non-medical graders performed screening for a population-based screening cohort at home. | Data drawn from 3 separate studies conducted in a single province of China 2014- 2019.  Two comparison cohorts received general assessment/ DR screening by Ophthalmologist  Group 1: Secondary-level passive case-finding cohort  Group 3: Population cohort, patients received ocular examinations with dilation of the pupil at home and screened for DM using point-of-service HbA1c testing |
| Mohan, 2012  India  Quality: 7 | Who: Diabetes technicians/village health workers, ophthalmologist, diabetologist, dietitians, podiatrist  Where: 42 villages in and around Chunampat, Chithamur block, Kancheepuram district, Tamil Nadu  Diabetes hospital in Chennai, Tamil Nadu | Model of care combining awareness/education campaign, outreach screening for diabetes including blood glucose testing, DR imaging by local staff sent via telemedicine to hospital ophthalmologists, and coronary artery disease testing, urine sampling | Who: Not reported  Where: Not reported | Not reported |
| Amritanand, 2018, India  Quality: 7 | Who: Community health workers, trainers, ophthalmologist, optometrist and paramedical worker  Where: Rural and tribal service areas of the Department of Community Health of a tertiary teaching hospital in Tamil Nadu | Community health workers were trained to conduct house-to-house surveys to detect those with perceived visual impairment. Then special temporary ophthalmic clinics were set up in a school within the screening area. The ophthalmologist performed a comprehensive eye examination and referred to the base hospital as required. | Who: primary eye care workers (paramedical ophthalmic assistants) and ophthalmologists  Where: Rural and tribal service areas of the Department of Community Health of a tertiary care teaching hospital in Tamil Nadu | Patients who self-present or referred by a health worker (outside of the selected intervention areas) to the regular peripheral clinics. |
| Katibeh, 2020, Iran  Quality: 7 | Who: Primary healthcare workers (PHCWs) and ophthalmic technician  Where: Varamin, Qarchak, Pishva, Pakdasht districts in Tehran province. | The local PHCWs went door-to-door and conducted needs assessments and vision **tests at the residence using a mobile application** (Peek Acuity and Peek Retina). Those requiring a retina evaluation were referred to the local primary healthcare facility to have mydriasis and fundus video captured by the trained technician. Those with presenting visual acuity worse than 6/12 in either eye were referred to an ophthalmologist. | Who: Primary healthcare workers and ophthalmic technician  Where: Varamin, Qarchak, Pishva, Pakdasht districts in Tehran province. | The local PHCWs went door-to-door and **referred them to the primary healthcare facility** for a visual acuity screening and fundus imaging. Those with presenting visual acuity worse than 6/12 in either eye were referred to an ophthalmologist. |
| Chariwala, 2020, India  Quality: 6 | Who: Accredited Social Health Activists (ASHAs)  Where: Communities around the community health centre, South Gujarat | **Group 2 – Phase 1**   - ASHAs were trained at the base hospital to educate people on DM and DR using the printed information‑education‑communication (IEC).   **Group 2 – Phase 2**  The same ASHAs were given case‑specific incentive for PwDM facilitated for DR screening. | Who: None  Where: Community health centre, South Gujarat | **Group 1:**   - Baseline of usual care   Screening of PwDM reporting at the community health centre |
| Raman, 2011  India  Quality: 6 | Who: Social worker, optometrist, retinal specialist  Where: Six villages of Tamil Nadu | A rural telehealth program for diabetic retinopathy (visual acuity and fundus photography) run as outreach camps by an optometrist who transferred real-time images to a specialist at the base hospital to confirm DR presence and grading. | Who: Not reported  Where: Not reported | Not reported |
| Adepoju, 2022, Nigeria  Quality: 5 | Who: medical, optical and surgical services  Where: 65 communities across Ilorin, Kwara State | At each community outreach, a nurse registers all the people to be screened. A specially designed clerking sheet is used by the resident doctors or consultants for screening and treatment. After each outreach visit, the information recorded on the pro forma are transferred into an outreach register. The outreach provides a comprehensive eye care involving medical, optical, and surgical services. The operated patients routinely have three follow-up contacts post-surgery. | Who: Not reported  Where: Not reported | Not reported |
| Murthy, 2020  India  Quality: 5 | Who: National Task Force, mentoring partners, technical expert groups, physicians, ophthalmologists, paramedical ophthalmic assistants, health support personnel  Where: 10 district hospitals in 10 states across India, | Integrating DR clinics into the non-communicable disease clinics | Who: Ophthalmologist  Where: Any eye facility | A retinal examination is opportunistic when people with DM visit an eye facility |
| Sangameswaran 2016  Quality: 4 | Who: Ophthalmologists, theatre personnel, optometrist, ophthalmic assistant, camp administrator  Where: Schools and community halls in Tamil Nadu | Cataract surgery in a mobile eye surgical unit [MESU] that includes a preparatory (pre-operative procedures) and a surgery vehicle. The ophthalmologist, optometrist and ophthalmic assistant would return to the sites 1 week and 1 month postoperatively. | Who: Not reported  Where: Not reported | Screening camps to identify cataract that are then referred to base hospitals for surgery. |
| Khan, 2022  Pakistan  Quality: N/A | Who: School teachers, Lady Health Visitors(LHVs), optometrists, dispensing opticians and programme managers  Where: communities and schools in Talagang Tehsil, Chakwal district, Punjab province and Matiari district, Sindh province | School and community eye screenings using Peek Vision by school teachers and LHVs and referring individuals to primary, secondary or tertiary eye care services, as required.  Establish eye screening at basic health units, and integrate optometry services at rural health centres connected to the secondary hospitals. | Who: Ophthalmologists  Where: Secondary hospitals, Tehsil Headquarter (THQ) Hospitals. | Routine ophthalmology services. |
| Pereira, 2012  Timor-Leste  Quality: N/A | Who: Eye care nurse, Fo-Naroman Timor-Leste  Where: Sub-district community health centres | Eye care nurse conducting outreach activities including vision screening, spectacle dispensing, health promotion, and referring those outside the scope of management. | Who: Eye care nurse  Where: Covalima District Hospital, Suai Town | Patients needing care would require travelling into the district hospital. |
| WHO 2023  Malaysia  Quality: N/A | Who: Ministry of Health, State hospital coordinating team,  Where: Sarawak, Kelantan, Pahang, Terrenganu states | KK-KKM Mobile: A bus that provides outreach cataract surgeries  KK-KKM Transit: Transports surgical equipment to district hospitals to perform cataract surgeries  KK-KKM Carnival: Mass surgeries on weekends at hospitals with specialist resources  KK-KKM Static: Redesigning tertiary hospital to be a one-stop centre for cataract surgery | Who: Not reported  Where: Not reported | Patients attend either public or private hospitals to have eye examinations and/or cataract surgery. |
| Awan 2018  Pakistan  Quality: N/A | Who: Lady Health Workers, Lady Health Supervisors, medical officers, optometrist, ophthalmic technician, ophthalmologists, data entry operator, diabetic educator  Where: Lahore District (Nishtar Town and Wagah Town) and Matiari District (Hala Taluka and Matiari Taluka) | 1. Training Lady Health Workers to build community awareness and refer individuals with or at risk of DM 2. Training medical officers to screen for DR at the Basic Health Units 3. DR Screening with optometrist at a Basic Health Unit and developed referral pathways to tertiary care where required   Develop a data management system | Who: Not reported  Where: Not reported | Not reported |
| ResInt 2019-2020  Bangladesh  Quality: N/A | Who: Not reported  Where: 4 governmental district hospitals, Ispahani Islamia Eye Institute & Hospital (IIEI&H), Nizam-Hasina Foundation (NHF), in Barishal and Bhola districts | Gender-friendly sensitisation workshops for eye care staff, separating eye hospital spaces (operation, waiting rooms) for gender-safe spaces, outreach camps, cascade training and strengthening referral systems (working with pharmacists). | Who: Not reported  Where: District Hospitals in Barishal division, Ispahani Islamia Eye Institute & Hospital (IIEI&H), Nizam-Hasina Foundation (NHF), Grameen G.C Eye Hospital, Barishal Diabetic Hospital, Patuakhali BNSB, Patuakhali Eye Hospitals and Dip Eye Hospital. | Patients who require eye care attend eye units at district hospitals or NGO-run eye care |

**S.5.2 TELEOPHTHALMOLOGY** **intervention description by quality score (n=10)**

| **Author, publication year, country** | **Provider and setting**  **(who/where)** | **Intervention content/method dose/frequency**  **(what, how, how often, how long)** | **Usual care provider and**  **setting (who/where)** | **Comparator/Usual care dose/frequency** |
| --- | --- | --- | --- | --- |
| De Araujo, 2021, Brazil  Quality: 11 | Who: Nurse Technicians and Remote Ophthalmologist  Where: Secondary Hospital | The nurse technician captured the images and shared off-site ophthalmologist remotely evaluates the images simultaneously or immediately after collection and may request the recapture of any images deemed unsatisfactory for reading.  The hospital where data collection was performed is equipped with identical remote examination rooms, which allowed both face-to-face and remote image acquisition to be performed simultaneously; the participants simply moved from one room to the other according to the preset randomization plan. | Who: On-site Ophthalmologist  Where: Secondary Hospital | Both sets of data collection were performed on the same day, in random order, by applying a randomization table to the schedule of the day’s appointments so that some patients would undergo face-to-face data collection first, while others would undergo remote collection first. |
| Bob-Semple 2017  Uganda  Quality: 10 | Who: lead researcher who was an ophthalmology trainee  Where: a university hospital | Used smartphone camera to take eye fundus photograph for diabetes patients immediately after the usual equipment used for standard assessment and repeated photo if low quality | Who: medical retina fellow  Where: in same university hospital | Indirect ophthalmoscopy with a panophthalmoscope (gold standard) and usual care |
| McKenna, 2018  China  Quality: 9 | Intervention 1:  Who: Non-medical graders, ophthalmic nurses  Where: County-level facilities in rural Southern China and Zhongshan Ophthalmic Centre (tertiary hospital)  Intervention 2:  Who: Rural ophthalmologists  Where: County-level facilities in rural Southern China | Intervention 1:  After 1 week of training, ophthalmic nurses captured retinal images and forwarded to non-medical graders at a tertiary hospital to assess image quality, grade the severity of DR/DMO.  Intervention 2:  Trained rural ophthalmologists’ assessment of the severity of DR/DMO status was determined by complete bilateral eye examinations on all patients aged 40 years and above, including all persons known to have DM. | Who: Retinal specialist  Where: Zhongshan Ophthalmic Centre | Assessing the image quality and grade the severity of DR/DMO from captured retinal images. |
| Yusuf 2022  Uganda  Quality: 8 | Who: Ophthalmologist, ophthalmic clinical officers  Where: Diabetic Clinic of Department of Medicine, Kiruddu National Referral Hospital | Regular eye examination with fundus dilation followed by a video captured with PEEK retina software, accessories and with an Android handheld smartphone. | Who: Ophthalmologist, ophthalmic clinical officers  Where: Diabetic Clinic of Department of Medicine, Kiruddu National Referral Hospital | Regular eye examination with fundus dilation followed by a picture of the posterior pole captured with the Zeiss Visucam 200 ophthalmic fundus camera. |
| Queiroz, 2020, Brazil  Quality: 7 | Who: Nurses and remote ophthalmologist  Where: basic health units (UBSs) in Sao Paulo | Using a smartphone-based handheld device, anterior and posterior ocular segment images were taken after mydriasis that was store-and-forwarded to the remote ophthalmologist for assessment. Continuous remote feedback was provided by the ophthalmologist to the nurses. | Not applicable | No control group |
| Collon, 2020, Nepal  Quality: 7 | Who: Ophthalmic technician and remote ophthalmologist  Where: Districts of Solukhumbu, Dolakha and Ramecchap | Patients go through registration, visual acuity testing, then an examination with a pen light, direct ophthalmoscope, retinoscope and handheld portable slit lamp. Pupils were then dilated (if not already) and anterior segment and central fundus photos/videos were captured with a handheld smartphone device. The images/video was forwarded to the remote ophthalmologists for recording diagnosis. | Who: Ophthalmic technician  Where: Districts of Solukhumbu, Dolakha and Ramecchap | Patients go through registration, visual acuity testing, then an examination with a pen light, direct ophthalmoscope, retinoscope and handheld portable slit lamp. Diagnosis for each eye was then recorded |
| Das, 2019,  India  Quality: 7 | Who: LV Prasad Eye Institute (LVPEI)  Where: 4 states (Andhra Pradesh,  Telangana, Karnataka, and Odisha) of India | EyeSmart EMR is an in-house electronic medical record system to provide Tele-ophthalmology and video calling at vision center. The vision center is equipped with vision testing, spectacle prescription, and slit lamp examination.  The Vision Technician connect with the Ophthalmologist at the command center. Consultation is provided in real time and the patient is guided and referred to the next level facility for further treatment. | Not applicable | No control group |
| Ortiz-Basso, 2022  Argentina  Quality: 7 | Who: Not reported  Where: Rural in La Pampa Argentina | A teleophthalmology program (the post-intervention period) for patients with diabetes in 2019 classified images from the disease register as referrable or not referrable based on severity and the former are seen by a specialist. | Who: ophthalmologists among others not specified  Where: Urban areas in the same province. | ‘traditional’ medical attention with fundus eye test (the before program implementation period) in 2018 |
| Keshvardoost, 2020, Iran  Quality: 4 | Who: Photographer, retinal specialists and general practitioners  Where: Tertiary hospital, Shafa Hospital and Basir subspecialty | Retinal images were taken, store-and-forwarded (stored for 2 months) for interpretation by retinal specialist and general practitioners. Retinal specialists graded the severity of diabetic retinopathy and presence of macula oedema while general practitioners graded normal (non-referral) / abnormal (referral). | Who: Retinal specialist  Where: Tertiary hospital, Shafa Hospital and Basir subspecialty | Face-to-face diabetic retinopathy examination with retinal specialists. |
| John, 2015  India  Quality: 1 | Who: Optometrists, social workers, administrative staff, ophthalmologists, IT experts  Where: Remote villages in Kanchipuram and Thiruvallur districts in Tamil Nadu, and Sankara Nethralaya Hospital | Outreach bus equipped with ophthalmic equipment for comprehensive eye examinations and telehealth with a hospital-based ophthalmologist to provide consultation treatment advise. Referred patients were provided with free surgery. | Who: Not reported  Where: Not reported | Not reported |

**S.5.3 HEALTH FINANCING intervention description by quality score (n=6)**

| **Author, publication year, country** | **Provider and setting**  **(who/where)** | **Intervention content/method dose/frequency**  **(what, how, how often, how long)** | **Usual care provider and**  **setting (who/where)** | **Comparator/Usual care dose/frequency** |
| --- | --- | --- | --- | --- |
| Essue 2020,  Viet Nam  Quality: 11 | Who: Payer assumed to be Government or Facility as a public payer or a development assistance partner  Where: N/R assumed in hospitals | Program A: eliminating all medical out-of-pocket cots for small incision cataract surgery  Program B: Program A + a voucher programme covering non-medical out-of-pocket costs. | Who: Presumed Vietnam Government Health insurance program | Patients pay medical and non-medical out-of-pocket cost associated with cataract surgery |
| Chen, 2011, China  Quality:10 | Who: Chinese Government  Where: 4 tertiary and 4 secondary hospitals in Yuzhong, Shapingba, Jiangbei, Jiulongpo, Dadukou and Nanan districts of Chongqing province. | In addition to the initial Urban Employee Basic  Medical Insurance Program,  the New Cooperative Medical Scheme (for rural populations) and Urban Resident Basic Health Insurance Scheme were available to Chinese citizens to be covered for cataract surgery. | Who: Chinese Government  Where: 4 tertiary and 4 secondary hospitals in Yuzhong, Shapingba, Jiangbei, Jiulongpo, Dadukou and Nanan districts of Chongqing province. | Urban Employee Basic Medical Insurance Program was available to Chinese citizens to be covered for cataract surgery. |
| Huang, 2012, China  Quality: 10 | Who: Doctors and study interviewers  Where: Two rural hospitals in Yunan and Qujiang counties of Guangdong province | Eligible patients who had cataract surgery were invited for the 3-months post-surgical study examination through television advertisements, posters, and a phone call to individual households whenever possible. They were informed the transportation costs for attending would be compensated for 50RMB. | Who: Not reported  Where: Not reported | Not assessed in this study but assumed to be just doctors advising the patients to return 3-months post-surgery. |
| Kessy 2007  Tanzania  Quality: 7 | Who: NR (presumably clinician authors)  Where: Referral University hospital in Tanzania | All patients who had failed to return for surgery after diagnosis were counselled about surgery, the cost, and the possibility of getting a waiver from the village leader if they could prove their inability to pay. | Not applicable | No control group |
| Okoye, 2015, Nigeria  Quality: 5 | Who: NR (presumably hospital managers)  Where: Ophthalmology unit at the University of Nigeria Teaching Hospital public, | Cataract surgery fees reduced in response to low surgery uptake by population (monitored over 2 years):  Adults: NGN 18,000.00  Children: NGN 15,000.00  Outreach patients: NGN 10,000.00 | Who: N/R  Where: Ophthalmology unit at the University of Nigeria Teaching Hospital (UNTH) | Cataract surgery fees stayed the same as before (monitored over 2 years):  Adults: NGN 55,000.00  Children: NGN 60,000.00  Outreach patients: NGN 55,000.00 |

**S.5.4 HEALTH EDUCATION intervention description by quality score (n=5)**

| **Author, publication year, country** | **Provider and setting**  **(who/where)** | **Intervention content/method dose/frequency**  **(what, how, how often, how long)** | **Usual care provider and**  **setting (who/where)** | **Comparator/Usual care dose/frequency** |
| --- | --- | --- | --- | --- |
| Ko, 2021  Myanmar  Quality: 10 | Who: Health educators  Where: Villages in Lewe Township, Naypyitaw Union Territory | Door-to-door health education was conducted by the trained health educators once a month for three consecutive months using specifically designed materials on blindness and eye diseases in terms of prevention and treatment, as well as availability, accessibility, and safety of the services. The topics included types of cataracts, awareness of cataracts, poor vision among the elderly, preventive measures, and the role of eyecare to prevent cataracts. Videos, handouts, education, and communication (IEC) materials such as pamphlets, T-shirts, posters, and calendars with health messages were provided. | Who: Not reported  Where: Villages in Lewe Township, Naypyitaw Union Territory | One-time group health education session that focussed on treatment options for cataracts and locations for treatment. |
| Liu, 2012, China  Quality:9 | Who: Ophthalmic nurse and on-site ophthalmologist  Where: Six county-level hospitals in Guangdong province. | Ophthalmic nursed measured presenting visual acuity followed by an ophthalmologist examining ocular health (without dilation). Those randomized to the intervention were provided:  A 5-minute **informational video** about cataract and cataract surgery, played using project and speakers in a darkened room separate from the examination area. This was followed by a nurse providing a 5-minute **counselling session**. | Who: Ophthalmic nurse and on-site ophthalmologist  Where: Six county-level hospitals in Guangdong province. | Ophthalmic nursed measured presenting visual acuity followed by an ophthalmologist examining ocular health (without dilation). Eligible patients (with cataract) were advised they had decreased vision due to cataract and that it could be treated with surgery. |
| Ramagiri, 2020, India  Quality: 6 | Who: Not reported  Where: Urban slums of Hyderabad | Group 1: Patients with DM were provided pamphlets or read to about DR  Group 2: Patients with DM were shown a locally made video about DR. They were shown in schools, community halls and Anganwadi centres. | Who: Not reported  Where: Not reported | Not reported |
| ResInt 2019,  Bangladesh  Quality:N/A | Who: nurses, trained technicians, paramedics medical officers, ophthalmologists  Where: Barishal district | 1. Community awareness campaigns on DR, diabetes and available services through local media, message boards, and community areas. This was followed by a DR screening camp 2. Basic eye care training was also provided to community members   Integrating diabetic eye care into primary or secondary health care systems and developing structured referral pathways. | Who: Not reported  Where: Not reported | Not reported |

**S.5.5 DIGITAL INNOVATION and ARTIFICIAL INTELLIGENCE intervention description by quality score (n=3)**

| **Author, publication year, country** | **Provider and setting**  **(who/where)** | **Intervention content/method dose/frequency**  **(what, how, how often, how long)** | **Usual care provider and**  **setting (who/where)** | **Comparator/Usual care dose/frequency** |
| --- | --- | --- | --- | --- |
| Mathenge et. al, 2022  Rwanda  Quality: 10 | Who: Diabetologist and other clinic staff  Where:  at 4 diabetes clinics in and around (2 district-level clinics, 1 referral-level clinic, and 1 diabetes association-led clinic). | Screening using retinal imaging with AI interpretation during routine visits  Intervention participants   - Were aware that their screening report was automatically generated by the AI platform - group was aware that their images would also be reviewed by human graders - received a report that included their fundus images and was colour coded for severity of the DR grade (green, no DR; yellow, mild DR; orange, moderate DR; and red, vision threatening DR). - were informed that referral to a secondary clinic for further ocular examination was required. | Who: Clinic staff unspecified  Where:  Participants in the control group (human grading) attended the same clinics.  Uptake of referrals within 30 days of receiving positive screening results were monitored. | Control participants: examined during routine visits were unaware of the AI report for others but aware that they would be contacted about whether to follow-up at the eye clinic through short message service (SMS) and also through a phone call from the health worker who attended them, after the human grading report was completed in 3 to 5 days.  Only after screeners had received human grading reports were control participants informed that they needed to visit the referral clinic. |
| Natarajan, 2019, India  Quality: 10 | Who: Staff from various dispensaries with no prior experience used the camera. Reports by AI.  Where: Municipal Corporation of Mumbai, India | A smartphone-based retinal camera and image analysis using Artificial Intelligence system to capture fundus images for offline, followed by automated analysis. Operators took additional pictures if AI system flagged them as poor quality. | Who: health care worker and ophthalmologist  Where: various dispensaries (primary healthcare facilities) | The images captured by Ophthalmologists were investigated through AI based system to ascertain accuracy against the human gold standard |

**S6. Detailed results for low quality and non-assessable studies**

**Table S6.1 Low quality outreach studies**

| **Author/year** | **Target condition** | **Quality score / 12** | | **REACH: Did it include**  **intended group or enhance**  **access to…? (Y/N/-)** | | | | | **Effectiveness** | | **Maintenance** |  |
| --- | --- | --- | --- | --- | --- | --- | --- | --- | --- | --- | --- | --- |
|  |  |  |  | **Women Rural Older** | | | | |  |  |  |  |
| Raman 2011 | DR | 6 | | - | | Y | Y | | | **73.6% (95%CI 70-77)** of the rural diabetics had never undergone eye examination previously. Mean age of those reached 53.8 years  **68%** (19/28) of those referrals reported for comprehensive eye evaluation at the base hospital.  Agreement between the presence and absence of DR was good **(K =79%)**. But agreement on severity level was low (ICC=0.76) | **NR** | |
| Chariwala 2020 | ?GO | 6 | | N | | - | N | | Screening increased from baseline the ASHA worker incentive group (RR **4.37, 95%** confidence interval (CI) **2.79, 6.84**] and  HE group without incentive (RR = **3.67, 95% CI 2.35, 5.75**). p NS between groups | | **N** | |
| Murthy 2020 | DR | | 5 | | - | - | | - | | **Seven-fold increase** in DM screening after integration of screening and management into public health system (rural areas included in overall estimate).  16.2% DR detected; 7.5% sight threatening DR; **7.6-fold increase** in treatments. Only 10.1% returned for next annual assessment | **NR** | |
| Adepoju 2022 | GO | | 5 | | - | Y | | Y | | 38% of all diagnosed with cataract underwent surgery.  2.14% individuals with refractive error had surgery or accepted glasses.  58% of patients were **aged >40 years** | **N** | |
| Sangameswaran 2016 | C | | 4 | | Y | Y | | Y | | The mobile cataract surgical unit reached 2,021 in remote locations, **mean age 61,4 years** (**53.6%** women)  Post surgical visual acuity of 6/9 or better was similar in men (37%) and women (42.3%), p=0.247 | **Y** | |

**Table S.6.2 No quality ranking - Outreach studies from reports in the grey literature**

| **Author/year** | **Target condition** | **Quality score / 12** | **REACH: Did it include**  **intended group or enhance**  **access to…?** | | | | **Effectiveness** | **Maintenance** |  |
| --- | --- | --- | --- | --- | --- | --- | --- | --- | --- |
|  |  |  | **Women Rural Older** | | | |  |  |  |
| Pereira 2013 | C/RE | N/A | | Y | - | - | Combining hospital consultations (government) and outreach (NGO), 3.5 times as many people were screened compared to 12 months prior. Women’s participation in outreach screening was statistically significantly higher than at hospital χ^2^=16.2 p<0.0001 | **Y** | |
| WHO 2023 | C | N/A | | - | Y | - | The mobile cataract surgical bus achieved **2.5-fold increase** in screening and surgery rates over 7 years, performing 21,003 operations |  | |
| Khan 2022 | GO | N/A | | Y | - | - | Screening coverage improved from **774/mth to 28,300/mth**.  Women participated in screening more than men (66.9-81.9%).  Referral adherence for women also improved **from 45% (95CI 42-48% in 2019) to 78% (95CI 76-80% in 2020)** and 92% of those referred were true positives for treatment need.  Reduction of false positive referral rate through retraining (from 86% to 17%) in one facility | **Y** | |
| ResInt 2019 | DR | N/A | | - | - | - | **75%** referred patients received treatment in the tertiary hospitals.  Of those referred to district hospitals **77%** claimed to have continued their treatment in the district hospital.  No differences in access by gender or rurality. | **Y** | |
| Awan 2018 | DR | N/A | | Y | Y | - | Diabetes risk assessment: The number of males screened for DR increased by 32.2% compared to an increase in women of **94.6%**  Basic Health Unit screening: The number of males increased by 61.2% compared to a lesser increase in women of **50.9%** | **Y** | |

*C= Cataract RE= refractive error DR=Diabetic retinopathy GO=General Ophthalmology*

*Y=Yes N=No P=Possibly NGO=non-government organisation*

** Blank cells (-) indicate not reported; absent p values indicate not reported or lack of statistical comparison.*

**Table S.6.3 Low quality Teleophthalmology studies**

| **Author/year** | **Target condition** | **Quality score / 12** | **REACH: Did it include**  **intended group or enhance**  **access to…?** | | | **Effectiveness** | **Maintenance** |
| --- | --- | --- | --- | --- | --- | --- | --- |
|  |  |  | **Women Rural Older** | | |  |  |
| Keshvardoost 2020  Iran | DR | 4 | - | - | - | Sensitivity for DR referrals from specialists was 85-92%, and for GPs were 93-95%; Diagnosis rate >90%. Estimate of avoidable referrals ranged from 40-55%  Agreement between GPs and specialists in their diagnosis was 0.88 – 0.91.(good to avert referrals)  67.5% of participants were female | **NR** |
| John, 2015  India | C | 1 | N | Y | - | 15% of telehealth examined patients with real-time hospital-based specialist advice had cataracts and were immediately transferred to city hospitals for free surgery.  48.7% of those screened were female. | **NR** |

*C= Cataract DR=Diabetic retinopathy NR= Not reported*

*Y=Yes N=No*

**Table S.6.4 Low quality Health financing studies**

| **Author/year** | **Target condition** | **Quality score / 12** | **REACH: Did it include**  **intended group or enhance**  **access to…?** | | | **Effectiveness** | **Maintenance** |
| --- | --- | --- | --- | --- | --- | --- | --- |
|  |  |  | **Women Rural Older** | | |  |  |
| Okoye 2015  Nigeria | C | 5 | N | - | N | The average annual uptake of cataract surgery was 94 overall, 82 pre and 106 post despite surgical fee reductions  no difference in uptake by gender at either period p=0.8337 | **NR** |

*C= Cataract DR=Diabetic retinopathy NR= Not reported*

*Y=Yes N=No*

**Table S.6.5 Low quality or non-assessable Health education studies**

| **Author/year** | **Target condition** | **Quality score / 12** | **REACH: Did it include**  **intended group or enhance**  **access to…?** | | | **Effectiveness** | **Maintenance** |
| --- | --- | --- | --- | --- | --- | --- | --- |
|  |  |  | **Women Rural Older** | | |  |  |
| Ramagiri 2020  India | DR | 5 | N | - | N | The average annual uptake of cataract surgery was 94 overall, 82 pre and 106 post despite surgical fee reductions  no difference in uptake by gender at either period p=0.8337 | **NR** |
| ResInt 2019-20  Bangladesh | DR | N/A | N | - | NR | Uptake of DR screening 2 months following education: Overall  OR 2.9 (1.48–5.52, P = 0.0018)].  After education through videos:  31.7% women vs 34.1% men p= NS  After receiving pamphlets:  14.5% women vs 7.3% men ?p=NR | **NR** |

*C= Cataract DR=Diabetic retinopathy NR= Not reported*

*N/A=not assessable for risk of bias Y=Yes N=No NS= not statistically significant*

**S7 Preferred Reporting Items for Systematic reviews and Meta-Analyses extension for Scoping Reviews (PRISMA-ScR) Checklist**

| **SECTION** | **ITEM** | **PRISMA-ScR CHECKLIST ITEM** | **REPORTED ON PAGE #** |
| --- | --- | --- | --- |
| **TITLE** | | | |
| Title | 1 | Identify the report as a scoping review. | 1 |
| **ABSTRACT** | | | |
| Structured summary | 2 | Provide a structured summary that includes (as applicable): background, objectives, eligibility criteria, sources of evidence, charting methods, results, and conclusions that relate to the review questions and objectives. | 1 |
| **INTRODUCTION** | | | |
| Rationale | 3 | Describe the rationale for the review in the context of what is already known. Explain why the review questions/objectives lend themselves to a scoping review approach. | 2 |
| Objectives | 4 | Provide an explicit statement of the questions and objectives being addressed with reference to their key elements (e.g., population or participants, concepts, and context) or other relevant key elements used to conceptualize the review questions and/or objectives. | 3 |
| **METHODS** | | | |
| Protocol and registration | 5 | Indicate whether a review protocol exists; state if and where it can be accessed (e.g., a Web address); and if available, provide registration information, including the registration number. | 4 |
| Eligibility criteria | 6 | Specify characteristics of the sources of evidence used as eligibility criteria (e.g., years considered, language, and publication status), and provide a rationale. | 3 |
| Information sources* | 7 | Describe all information sources in the search (e.g., databases with dates of coverage and contact with authors to identify additional sources), as well as the date the most recent search was executed. | 3 |
| Search | 8 | Present the full electronic search strategy for at least 1 database, including any limits used, such that it could be repeated. | Supp 1 |
| Selection of sources of evidence† | 9 | State the process for selecting sources of evidence (i.e., screening and eligibility) included in the scoping review. | 4, Supp 3 |
| Data charting process‡ | 10 | Describe the methods of charting data from the included sources of evidence (e.g., calibrated forms or forms that have been tested by the team before their use, and whether data charting was done independently or in duplicate) and any processes for obtaining and confirming data from investigators. | 4 |
| Data items | 11 | List and define all variables for which data were sought and any assumptions and simplifications made. | 3-4 |
| Critical appraisal of individual sources of evidence§ | 12 | If done, provide a rationale for conducting a critical appraisal of included sources of evidence; describe the methods used and how this information was used in any data synthesis (if appropriate). | 4, Supp 2 |
| Synthesis of results | 13 | Describe the methods of handling and summarizing the data that were charted. | 4 |
| **RESULTS** | | | |
| Selection of sources of evidence | 14 | Give numbers of sources of evidence screened, assessed for eligibility, and included in the review, with reasons for exclusions at each stage, ideally using a flow diagram. | 4-5, Fig 1 |
| Characteristics of sources of evidence | 15 | For each source of evidence, present characteristics for which data were charted and provide the citations. | 5, Supp 4 |
| Critical appraisal within sources of evidence | 16 | If done, present data on critical appraisal of included sources of evidence (see item 12). | 5, Fig 2, Supp 5 |
| Results of individual sources of evidence | 17 | For each included source of evidence, present the relevant data that were charted that relate to the review questions and objectives. | combined |
| Synthesis of results | 18 | Summarize and/or present the charting results as they relate to the review questions and objectives. | 6-11, T2-6 |
| **DISCUSSION** | | | |
| Summary of evidence | 19 | Summarize the main results (including an overview of concepts, themes, and types of evidence available), link to the review questions and objectives, and consider the relevance to key groups. | 11-13 |
| Limitations | 20 | Discuss the limitations of the scoping review process. | 13 |
| Conclusions | 21 | Provide a general interpretation of the results with respect to the review questions and objectives, as well as potential implications and/or next steps. | 14-15 |
| **FUNDING** | | | |
| Funding | 22 | Describe sources of funding for the included sources of evidence, as well as sources of funding for the scoping review. Describe the role of the funders of the scoping review. | 15 |

JBI = Joanna Briggs Institute; PRISMA-ScR = Preferred Reporting Items for Systematic reviews and Meta-Analyses extension for Scoping Reviews.

* Where *sources of evidence* (see second footnote) are compiled from, such as bibliographic databases, social media platforms, and Web sites.

† A more inclusive/heterogeneous term used to account for the different types of evidence or data sources (e.g., quantitative and/or qualitative research, expert opinion, and policy documents) that may be eligible in a scoping review as opposed to only studies. This is not to be confused with *information sources* (see first footnote).

‡ The frameworks by Arksey and O’Malley (6) and Levac and colleagues (7) and the JBI guidance (4, 5) refer to the process of data extraction in a scoping review as data charting*.*

§ The process of systematically examining research evidence to assess its validity, results, and relevance before using it to inform a decision. This term is used for items 12 and 19 instead of "risk of bias" (which is more applicable to systematic reviews of interventions) to include and acknowledge the various sources of evidence that may be used in a scoping review (e.g., quantitative and/or qualitative research, expert opinion, and policy document).

*From:* Tricco AC, Lillie E, Zarin W, O'Brien KK, Colquhoun H, Levac D, et al. PRISMA Extension for Scoping Reviews (PRISMAScR): Checklist and Explanation. Ann Intern Med. 2018;169:467–473. [doi: 10.7326/M18-0850](http://annals.org/aim/fullarticle/2700389/prisma-extension-scoping-reviews-prisma-scr-checklist-explanation).
